# Discovery of Mitochondrial Complex I Inhibitors as Anticancer and Radiosensitizer Drugs Based on Compensatory Stimulation of Lactate Release

**DOI:** 10.3390/cancers14215454

**Published:** 2022-11-06

**Authors:** Junjie Lan, Octavia Cadassou, Cyril Corbet, Olivier Riant, Olivier Feron

**Affiliations:** 1 Pole of Pharmacology and Therapeutics (FATH), Institut de Recherche Expérimentale et Clinique (IREC), Université catholique de Louvain (UCLouvain), 1200 Brussels, Belgium; 2 Institute of Condensed Matter and Nanosciences (IMCN), Molecular Chemistry, Materials and Catalysis (MOST), Université catholique de Louvain (UCLouvain), 1348 Louvain-la-Neuve, Belgium; 3 Walloon Excellence in Life Sciences and BIOtechnology (WELBIO) Department, WEL Research Institute, avenue Pasteur 6, 1300 Wavre, Belgium

**Keywords:** cancer, L-lactate, glycolysis, spheroid, respiration, mitochondria, complex I, oxygen consumption rate, radiotherapy, reoxygenation

## Abstract

**Simple Summary:**

To identify inhibitors of mitochondrial respiration as potential anticancer drugs is not an easy matter since cancer cells cultured as monolayers may escape by shifting their metabolic preference toward the use of glycolysis. Here we propose to capitalize on this apparent weakness to exploit the associated increase in L-lactate release as a primary screening assay to identify and optimize the development of inhibitors of mitochondrial oxidative phosphorylation. As a secondary assay, we used a protocol based on O_2_ consumption rate in permeabilized cancer cells to get further insights on a possible direct or indirect inhibition of mitochondrial electron transport chain. Finally, 3D tumor spheroids helped us to further select drug candidates endowed with the capacity to exert growth inhibitory effects in tumor-mimicking conditions but also to act as potent radiosensitizers by promoting reoxygenation.

**Abstract:**

Cancer cells may stimulate glycolytic flux when O_2_ becomes insufficient. Increase in L-lactate release therefore appears as an escape mechanism to drugs targeting mitochondrial respiration but also represents a response that may be exploited to screen for compounds blocking either mitochondrial carriers of oxidizable substrates or the electron transport chain. Here, we developed a screening procedure based on the capacity of cancer cells to release L-lactate to gain insights on the development of mitochondrial complex I inhibitors. For this purpose, we synthesized derivatives of carboxyamidotriazole, a compound previously described as a potential OXPHOS inhibitor. Two series of derivatives were generated by cycloaddition between benzylazide and either cyanoacetamides or alkynes. A primary assay measuring L-lactate release as a compensatory mechanism upon OXPHOS inhibition led us to identify 15 hits among 28 derivatives. A secondary assay measuring O_2_ consumption in permeabilized cancer cells confirmed that 12 compounds among the hits exhibited reversible complex I inhibitory activity. Anticancer effects of a short list of 5 compounds identified to induce more L-lactate release than reference compound were then evaluated on cancer cells and tumor-mimicking 3D spheroids. Human and mouse cancer cell monolayers exhibiting high level of respiration in basal conditions were up to 3-fold more sensitive than less oxidative cancer cells. 3D tumor spheroids further revealed potency differences between selected compounds in terms of cytotoxicity but also radiosensitizing activity resulting from local reoxygenation. In conclusion, this study documents the feasibility to efficiently screen in 96-well plate format for mitochondrial complex I inhibitors based on the capacity of drug candidates to induce L-lactate release.

## 1. Introduction

While inhibition of mitochondrial respiration has long been thought to be unsafe, recent findings have led to reconsider electron transport chain as a valid anticancer target [1,2,3]. Indeed, while cytosolic glycolysis is a major pathway supporting cancer cell growth under hypoxia as well as in the presence of oxygen (the Warburg effect), mitochondria also largely contribute to fulfill the bioenergetic and biosynthetic needs of growing cancer cells [4]. While a large variety of substrates including pyruvate, lactate, amino acids and fatty acids can supply the TCA cycle at different stages, they all eventually end up generating NADH and FADH2 that in turn support oxidative phosphorylation (OXPHOS) [5]. A variety of repurposed drugs are endowed with OXPHOS inhibitory activity but to which extent associated anticancer effects are directly derived from disruption of mitochondrial function is unclear. The best example is the antidiabetic drug metformin which is a weak OXPHOS inhibitor (OXPHOSi) but also an activator of the energy sensor AMPK which *per se* may account for antiproliferative effects [3,6]. In order to gain in specificity, mitochondrial targeting through conjugation with the triphenylphosphonium cation was also recently applied to the antimalarial drug atovaquone to exploit its capacity to block mitochondrial electron transfer chain [7,8]. Besides compounds exhibiting auxiliary OXPHOS inhibitory effects, *bona fide* mitochondrial complex inhibitors were developed and validated in different mouse models [9,10,11,12,13]. Among them, IACS-010759 is a specific complex I inhibitor [10] which is currently undergoing clinical trials (NCT03291938, NCT02882321).

Remarkably, because of the metabolic plasticity of cancer cells, inhibition of cell respiration may not necessarily lead to cell death. Indeed, cancer cells may adapt and shift their metabolic preferences towards glycolysis upon OXPHOS inhibition. Screening for tumors exhibiting (mutational) defects in glycolytic flux may thus represent a pre-requisite to identify cancer patients the most likely to respond to OXPHOSi [14]. Combination with radiotherapy is another option since OXPHOS inhibition may lead to tumor reoxygenation which in turn sensitizes cancer cells to the effects of ionizing radiations [2]. Radiotherapy is indeed well known to benefit from a local tumor increase in pO_2_ that stabilizes radiation-induced DNA damages. We actually recently reported how a mitochondrial pyruvate carrier (MPC) inhibitor by preventing pyruvate transport into the mitochondria, significantly reduced OXPHOS and rendered tumor exposure to radiations more detrimental [15].

To identify compounds able to block OXPHOS in a drug screening campaign is challenging but recent technological developments offer new perspectives. In the last decade, the development of respirometer adapted to handle 96 well plates, the Seahorse technology, has indeed made available the possibility to measure oxygen consumption rate (OCR) on living cells, and thereby to establish the inhibitory activity of a compound on OXPHOS and even to identify the targeted complex within the electron transport chain. Still, a pre-screening procedure is needed to reduce the list of potential hits to be tested for OCR modulation. As stated above, while inhibition of cell respiration does not necessarily lead to cell death, the consecutive stimulation of glycolysis may be exploited as such a primary assay since in these conditions, a rising lactate concentration gradient will promote its efflux to the extracellular compartment. Measurements of extracellular L-lactate may thus represent a well-suited high-throughput methodology to identify compounds able to block either mitochondrial carriers of oxidizable substrates or respiratory complexes, both having the potential to result in OXPHOS inhibition. This strategy is further supported by evidence documenting that L-lactate (and not pyruvate) is the final product of glycolysis including under aerobic conditions [16,17] and is oxidized as a respiratory substrate by a mitochondrial LDH [18,19,20,21,22].

Another hurdle in the in vitro search for OXPHOSi is the need to integrate the hypoxic and more oxygenated cancer cell phenotypes that co-exist in vivo to have a better understanding of the resulting activity of potential hits. While hypoxia workstation allows to handle cancer cells under hypoxia, potential metabolic reoxygenation will be rapidly suppressed because of the imposed low pO_2_. On the other hand, mouse tumors do not offer the necessary throughput for the screening of OXPHOSi candidates. An attractive option therefore consists of the use of 3D cultures of cancer cells such as tumor spheroids wherein hypoxia spontaneously develops at some distance from the periphery [15]. These 3D models actually recapitulate the interdependence between cancer cells with distinct metabolic preferences as we previously reported with symbiotic L-lactate exchange [23,24,25]. The use of 3D spheroids may thus offer an additional downstream filter to determine whether physicochemical characteristics of selected compounds are compatible with their need to diffuse through several layers of cancer cells and go through plasma and mitochondrial membranes before reaching their target.

In this study, we have applied a multi-step screening procedure to identify mitochondrial complex I inhibitors and to rank them according to their therapeutic potential. For this proof of principle, we synthesized new derivatives of carboxyamidotriazole (CAI), a compound initially reported to reduce the calcium buffering capacity of mitochondria [26] through the dissipation of mitochondrial membrane potential before being documented as a direct OXPHOSi [27]. Along the pharmacomodulation process, we identified potential hits based on their capacity to induce L-lactate release that we further validated using Seahorse-based OCR measurements. Finally, the evaluation in 3D tumor spheroids led us to identify compounds with a higher anticancer activity than reference compound CAI and a higher radio-sensitizing potential.

## 2. Materials and Methods

### 2.1. Synthesis

Unless otherwise mentioned, reagents were purchased from commercial sources (Sigma Aldrich, Merck, Hoeilaart, Belgium; TCI, Zwijndrecht, Belgium; Acros, ThermoFischer scientific, Geel, Belgium; FluoroChemCompany, Amsterdam, The Netherlands) and used without further purification. All solvents were dried from NaH or CaH_2_ and purified by distillation before being used. NMR spectra were recorded at room temperature on a Bruker Avance UltraShield instrument operating at a frequency of 300 MHz for ^1^H and 75 MHz for ^13^C. Chemical shifts (*δ*) are reported in ppm relative to CDCl_3_ (*δ* = 7.26 ppm) for ^1^H NMR and CDCl_3_ (*δ* = 77.2 ppm) and DMSO-*d_6_* (*δ* = 2.50 ppm) for ^1^H NMR and CDCl_3_ (*δ* = 77.2 ppm) and DMSO-*d_6_* (*δ* = 39.52 ppm) for ^13^C NMR multiplicity (s = singlet, d = doublet, t = triplet, m = multiplet, q = quartet, br = broad). Column chromatography was performed over ROCC Silica gel 60 (40–63 µm). Thin layer chromatography was performed on prepared thin layers precoated plates: Silica gel Merck 60 F254. The visualization of spots on TLC plates was done by UV light (254 nm or 365 nm) or KMnO_4_ solution staining. Mass spectra were recorded using an orbitrap Q Exactive Thermo Fisher spectrometer, which is a hybrid quadrupole-orbitrap mass spectrometer.

### 2.2. Cell Culture

Mouse colorectal CT26 and breast 4T1 cancer cells as well as human cervix cancer SiHa and breast MDA-MD-231 cancer cells were acquired from ATCC where they are regularly authenticated by short tandem repeat profiling. They were stored according to the supplier’s instructions and used within 6 months after resuscitation of frozen aliquots. Cancer cells were maintained in DMEM (Life Technologies, ThermoFischer scientific, Merebelke, Belgium) supplemented with 10% heat-inactivated FBS. All cell lines were tested for mycoplasma contamination with the MycoAlert™ Mycoplasma Detection kit (#LT07-318; Lonza, Verviers, Belgium) before being used. Cell integrity was determined by the Trypan Blue exclusion method (using Bürker chambers) while cell growth was assessed by using the Presto Blue reagent (Life Technologies) according to manufacturer’s instructions.

### 2.3. Metabolic Profiling

L-lactate concentration was measured in a semi-automated manner using CMA Microdialysis AB enzymatic assay and a CMA 600 analyzer (Aurora Borealis, Schoonebeek, The Netherlands) as previously described [15]. Briefly, cells (2 × 10^5^ cells per well; 3 wells/condition) were seeded in 12-well plates with 2 mL of their routine culture medium. After 24 h, medium was replaced by 500 µL of DMEM containing 10 mmol/L D-glucose and supplemented with 2 mmol/L L-glutamine and 10% dialyzed FBS (Sigma-Aldrich), in the presence of the compounds or vehicle (DMSO). The initial concentration of L-lactate in the experimental medium was also assessed by including control wells containing only cell culture medium (no cells) on each plate. After incubation for 24 h, extracellular media were collected and deproteinized by centrifugation (15 min, 10,000 rpm, 4 °C) in 10 kDa cut-off filter tubes (VWR, Avantor, Leuven, Belgium). Differences in L-lactate concentrations between control and treated cells were calculated and normalized by the protein content in each well and expressed in µmol/h/mg protein. Oxygen-consumption rate (OCR) was measured using the Seahorse XF96 plate reader as previously reported [15,28]. Briefly, cells (2 × 10^4^ cells per well) were first permeabilized with 1 nM Seahorse XF Plasma Membrane Permeabilizer (#102504-100; Agilent, Machelen, Belgium) and respiration was stimulated by adding 10 mM pyruvate, 5 mM malate, and 2 mM ADP. After baseline OCR determination, compounds were added (to inhibit complex I) followed by 10 mM succinate to fuel complex II and rescue the electron transport chain. In some experiments, the effects of selected hits on basal OCR were evaluated in intact cells (including in the presence of MCT1/2 inhibitor AR-C155858 [29] or methylpyruvate [15]). OCR data were normalized according to protein content in each well.

### 2.4. 3D Cultures

Spheroids were prepared by seeding 1500 cells/well in Ultra-Low Attachment 96-well plate (Corning, Merck, Hoeilaart, Belgium) in DMEM supplemented with 10% heat-inactivated FBS. Inhibitors were added on day 4 (mean diameter of spheroids: 300 µm) and renewed every 3 days. Spheroid growth was monitored using live-cell phase contrast microscope (Axio Observer, Zeiss, Zaventem, Belgium); spheroid area, density and morphology were measured using ImageJ software. Confocal fluorescence microscopy (LSM510, Zeiss, Zaventem, Belgium) was used for detecting pimonidazole immunostaining as previously reported [15]. Spheroids from a same experiment were imaged with the same gain and exposure settings. In some experiments, OXPHOSi treatment was combined with irradiation using a ^137^Cs γ-irradiator (IBL-637; CIS-BioInternational, Saclay, France) as previously described [15,30].

### 2.5. Statistical Analysis

Results are expressed as mean ± SEM of at least three independent experiments as indicated in the figure legends. Two-tailed unpaired Student t-test, one-way or two-way ANOVA tests (Bonferroni’s post-hoc test) were used where appropriate.

## 3. Results

### 3.1. Synthesis of Carboxyamidotriazole Derivatives

**Figure 1:**
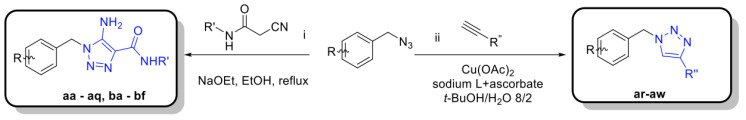
Main synthetic paths to generate carboxyamidotriazole derivatives. The key fragments benzyl azide, *N*-substituted-2-cyanoacetamides and alkynes were prepared by conventional synthetic methods. The 5-amino-4-carboxyamido-1,2,3-triazole derivatives were assembled by a 1,3-dipolar cycloaddition between 2-cyanoacetamide or *N*-substituted 2-cyanoacetamides with the corresponding benzyl azides, using sodium ethoxide as a base, as previously reported [31,32]. Another series of *N*-substituted 1,2,3-triazoles, lacking the amino group on the 5-position of the heterocycle, were also prepared by Huisgen-Click cycloaddition reaction between a suitable benzyl azide and an alkyne, using the standard copper acetate/sodium ascorbate system as a catalyst [33].

In order to set up a screening protocol to identify and/or optimize mitochondrial complex I inhibitors, carboxyamidotriazole (here below called reference compound **aa**) was modified so that 28 derivatives with different substituents were synthesized. A first series of derivatives were assembled by a 1,3-dipolar cycloaddition between 2-cyanoacetamide or *N*-substituted 2-cyanoacetamides with the corresponding benzyl azides (Figure 1, left). The second series of *N*-substituted 1,2,3-triazoles were prepared by cycloaddition reaction between a suitable benzyl azide and an alkyne (Figure 1, right). Structures of CAI derivatives are reported in Table 1; they were identified and analyzed by standard organic analytical techniques (see Supplementary Information).

### 3.2. Three-Step Procedure to Select for Mitochondrial Complex I Inhibitors as Anticancer Drugs

**Figure 2:**
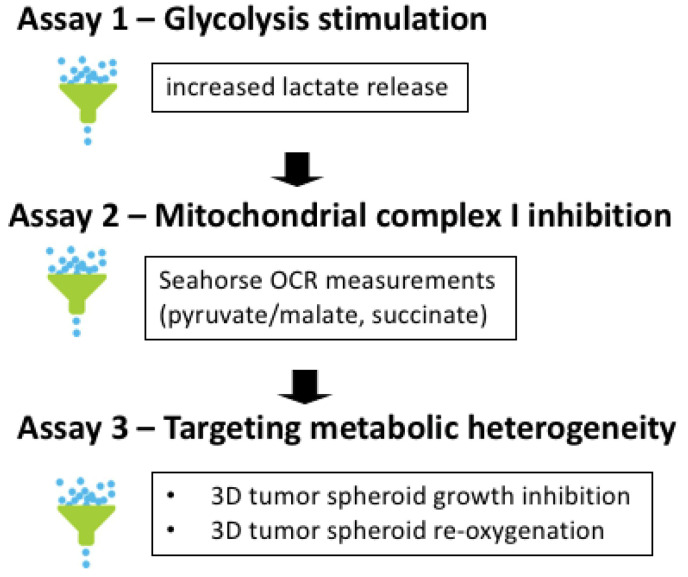
Screening procedure to identify anticancer activity of *bona fide* mitochondrial complex I inhibitors. The 3-step procedure includes the evaluation of (1) the stimulation of glycolysis upon OXPHOS inhibition (based on the increase in extracellular L-lactate release), (2) complex I inhibition (based on the measurement of real-time oxygen consumption rate (OCR) in permeabilized cancer cells exposed to a NADH-generating system and (3) the capacity to impact the growth of 3D tumor spheroids recapitulating tumor metabolic heterogeneity (through the determination of growth inhibitory effects, re-oxygenation and radio-sensitization).

We used a 3-step screening procedure to identify **aa** derivatives endowed with the capacity to act as complex I inhibitors in conditions mimicking the in vivo tumor microenvironment (Figure 2). Stimulation of glycolysis upon mitochondrial OXPHOS inhibition is a well-known observation [15], making increase in L-lactate release an attractive first screening step. Measuring real-time oxygen consumption rate (OCR) may then be used as a secondary assay to identify *bona fide* complex I inhibitor (Figure 2). This requires to expose permeabilized cancer cells to a mixture of pyruvate and malate as a NADH-generating system (fueling complex I) and then to evaluate the reversibility by adding succinate as a fuel of complex II [34]. Of note, the above anions which cannot permeate through mitochondrial membranes are transported down their gradients by carriers which operate non-electrogenic by anion exchange or proton symport [35,36,37]. The third step of our screening procedure aimed to test hit compounds in 3D tumor spheroids to recapitulate the tumor organization with the co-existence of both hypoxic and oxygenated cancer cells (Figure 2). This 3D cell culture set up is indeed more likely to reveal the impact of drugs with possible bystander effects.

### 3.3. L-Lactate Release Measurement as a Primary Assay to Identify Potential OXPHOS Inhibitors

**Figure 3:**
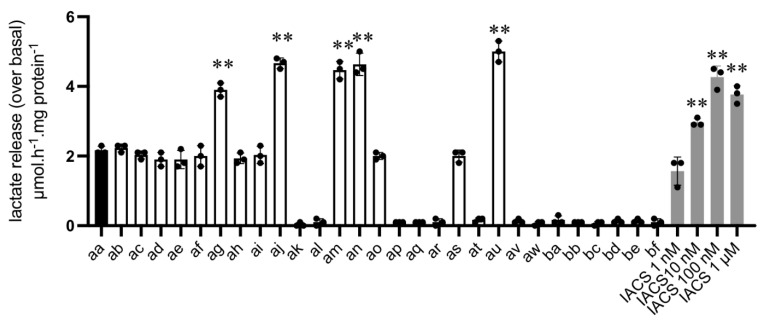
Compound-induced L-lactate release (Assay #1). Culture medium was collected after 24 h exposure to the indicated compounds (10 µM) and the amount of extracellular L-lactate release was determined in a semi-automated manner; dose-dependent effects of complex I inhibitor IACS are shown on the right (shaded bars). Bar graph depicts the extent of L-lactate secretion above the basal release as determined in the absence of any compound (n = 3, ** *p* < 0.01 for increased L-lactate release vs. reference compound **aa**).

As mentioned above, upon pharmacological inhibition of mitochondrial respiration, L-lactate will accumulate intracellularly and be consecutively released in the extracellular medium. Collection of culture medium after 24 h exposure to 10 µM **aa** derivatives and semi-automated measurements of L-lactate content led us to identify 15 compounds (including **aa** reference compound) endowed with the capacity to significantly stimulate L-lactate release from colorectal CT26 cancer cells (Figure 3). Among them, 9 were as active as **aa** and 5 were even more potent inducers of L-lactate release (**ag**, **aj**, **am**, **an** and **au**) (see Figure 3 and Table 2**,** assay 1). Interestingly, the highly potent complex I inhibitor IACS-010759 [10] stimulated L-lactate release to a similar extent to that observed in response to the 5 most active **aa** derivatives (see Figure 3). Similar results were obtained in breast cancer 4T1 cancer cells when comparing L-lactate release in response to **aa** compound vs. **au** and **at** compounds identified as more and less potent (than **aa**) in CT26 cancer cells, respectively (Supplementary Figure S1A).

### 3.4. Validation of Complex I Inhibitors Using Seahorse-Based OCR Measurements

**Figure 4:**
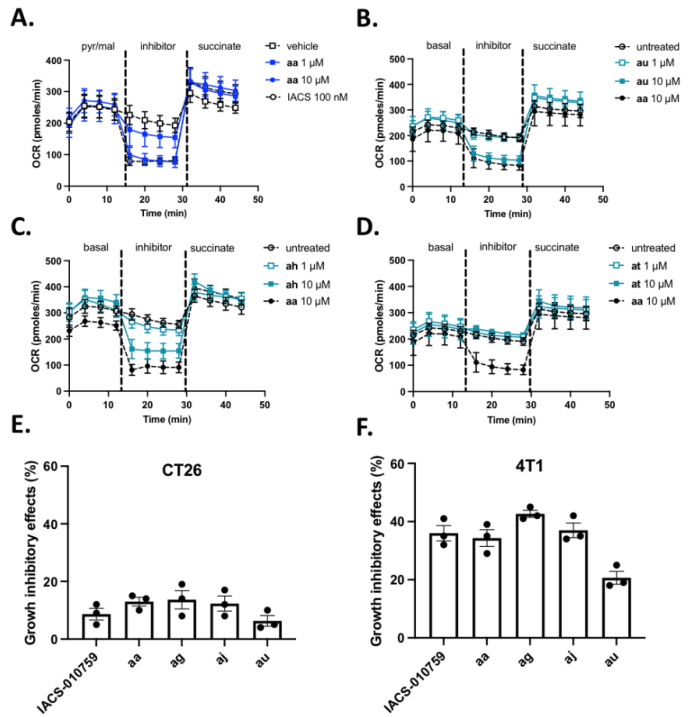
Mitochondrial complex I activity inhibition (Assay #2). (**A**–**D**)**.** Graphs depict changes in O_2_ consumption rate (OCR) measured with the Seahorse technology on permeabilized CT26 cancer cells upon successive addition of pyruvate/malate, the indicated inhibitor and succinate. OXPHOS I inhibitor IACS was used as a control in the experiments testing reference compound **aa** (**A**), and compound **aa** (10µM) was used as reference for testing **au, ah** and **at** derivatives (**B**–**D**) (n = 6 per compound). (**E**,**F**)**.** Growth inhibitory effects of cultured CT26 (**E**) and 4T1 (**B**) cancer cells exposed to 100 nM IACS or 1 µM **aa**, **ag**, **aj** and **au** compounds for 72 h; data represent the reduced extent (expressed as %) of cell viability (determined with Presto Blue) when compared with vehicle conditions (n = 3).

To establish a direct link between induction of L-lactate release and the capacity of **aa** derivatives to inhibit complex I, we then used the Seahorse technology that measures real-time oxygen consumption rate (OCR) from label-free cancer cells in a 96-well plate format. Using the protocol described above (i.e., sequential addition of pyruvate and malate, compounds and succinate in permeabilized cancer cells), we first compared the behavior of compound **aa** with complex I inhibitor IACS 10-759. We found that complex I-dependent OCR reduction in colorectal CT26 cancer cells was detectable at 1 µM aa and was maximal at 10 µM **aa** (i.e., the same extent of maximal inhibition as obtained with 100 nM IACS-010759) (Figure 4A). Similar results were obtained in permeabilized breast cancer 4T1 cancer cells (Supplementary Figure S1B).

Importantly, the full reversal of OCR inhibition upon succinate addition strongly supports the complex I inhibitory activity of **aa** compound which was so far described as an OXPHOSi on the basis of an indirect assay using a cocktail of mitochondrial proteins and NADH [27]. Moreover, in Seahorse experiments performed on (non-permeabilized) intact CT26 cancer cells exposed to **aa** compound, AR-C155858 used as an inhibitor of a putative mitochondrial MCT1/2 transporter [38] failed to restore OCR. Since other lactate transporters could be involved [39,40], we also used methylpyruvate which can bypass the need for a mitochondrial carrier and mimic the result of lactate oxidation by a mitochondrial LDH or ensure the delivery of pyruvate into the mitochondria matrix [15]. Methylpyruvate failed to restore OXPHOS activity, further supporting complex I inhibition and not the blockade of mitochondrial transporters as the most likely mechanism of action of **aa** compound. In the same experiments carried out on non-permeabilized cancer cells, the extent of basal OCR inhibition in response to 10 µM **aa** was similar to that observed with 100 nM IACS-010759 (−52 ± 5% and −45 ± 6%, respectively) and mirrored by an increase in extracellular acidification rate (ECAR) (+36 ± 11% and +29 ± 9%, respectively).

In our hands, the Seahorse technology requires to work with 6 replicates per condition to generate consistent measurements. To use it as a screening procedure with a reasonable throughput, we restricted the testing of **aa** derivatives to two concentrations (1 and 10 µM). Also, although the goal of the primary assay was to reduce the list of compounds of interest to those inducing an increase in L-lactate efflux, we have here tested all the compounds to identify possible false negative hits. Out of the evaluated 28 **aa** derivatives, different patterns of activity were observed (see representative patterns of equiactive (**au**), hypoactive (**ah**) and inactive compounds (**at**) (Figure 4B–D); the OCR inhibition patterns of all the compounds are provided as Supplementary Figure S2. Three hits were identified to be equiactive with **aa** with an IC_50_ below 10 µM, namely **ag**, **aj** and **au** (see also Table 2**,** assay 2). Eight other compounds (**ab**, **ac**, **ad**, **ae**, **af**, **ah**, **am** and **an**) inhibited complex I activity to a smaller extent than **aa** with an IC_50_ around 10 µM (i.e., the highest concentration tested) (Table 2**,** assay 2). All the other compounds did not significantly inhibit complex I activity (Table 2**,** assay 2). This secondary assay did not identify false negative compounds and actually confirmed 12 compounds out of the 15 hits identified thanks to the primary -based assay; only compounds **ai**, **ao** and **as** were able to promote L-lactate release but did not show significant inhibition of complex I even at 10 µM (compare assays 1 and 2 in Table 2).

It is important to note that 24 h-drug exposure in Assays 1 and 2 was used to unmask functional metabolic adaptation (i.e., stimulation of L-lactate release and OCR inhibition) and did not intend to induce major toxicity that could lead to confusing interpretation. Cell counting using Trypan blue exclusion was used to prove that the integrity of cancer cells remained unaltered 24 h after exposure to the tested compounds (90.9 ± 5.1% viable cancer cells after exposure to **aa** compound vs. 91.2 ± 4.2% after vehicle exposure). Still, to support the anticancer potential of complex I inhibition, we also examined the cytotoxic effects of longer exposure of intact cancer cells to reference compound **aa** and hit compounds **ag**, **aj** and **au**. Although very limited effects were observed after 72 h treatment of CT26 colon cancer cells (Figure 4E), a more significant growth inhibitory activity was detectable in breast 4T1 cancer cells (Figure 4F), in good agreement with the higher basal respiration (i.e., OXPHOS-dependency) of the latter (400 pmol·min^−1^ vs. 200–250 pmol·min^−1^ for CT26 cells, see Figure 4A–D and Supplementary Figure S1B). Larger antiproliferative effects of **aa**, **ag**, **aj** and **au** were also observed in oxidative human cervix Siha cancer cells when compared with highly glycolytic human breast MDA-MB-231 cancer cells (Supplementary Figure S3A,B). While the nature of cell death induced by **aa** derivatives is beyond the scope of this manuscript, alteration in mitochondria fitness and capacity to buffer calcium could be involved as previously reported [26].

### 3.5. Validation of the Cytotoxic and Radiosensitizing Potentials of aa Derivatives

**Figure 5:**
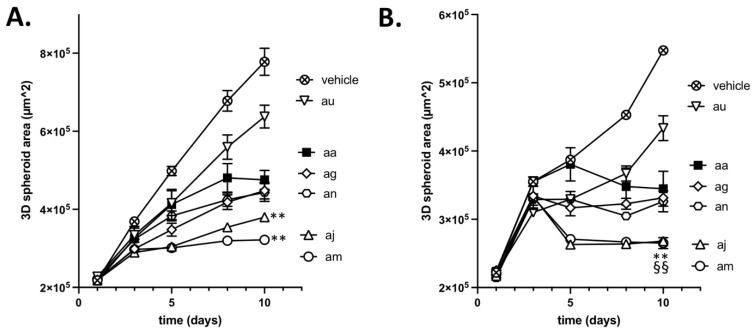
Inhibition of 3D tumor spheroid growth (Assay #3). Graphs depict (**A**) the direct growth inhibitory effects of the indicated compounds and (**B**) their capacity to exert radiosensitizing effects as determined using 3D tumor CT26 spheroids (n = 6 per condition). ** *p* < 0.01 for spheroid sizes at day 10 significantly smaller than spheroids treated with **aa** (**A**) or **aa** + irradiation (**B**) and ^§§^ *p* < 0.01 for spheroid sizes at day 10 significantly smaller than corresponding spheroids at day 3 (**B**). Drug exposure without (**A**) or with irradiation (**B**) was associated with a significant reduction in spheroid sizes vs. untreated spheroids (not shown).

The third and last step of our screening procedure aimed to further gain in knowledge of the most appropriate compound to be used in future in vivo evaluation. For this purpose, we evaluated in tumor-mimicking 3D spheroids the cytotoxicity of the above 3 hit compounds (**ag**, **aj** and **au**) and the 2 hypoactive compounds with however a higher capacity to induce L-lactate release (**am** and **an**). The profile of spheroid growth reveals that compounds **aj** and **am** were significantly more active than reference compound **aa** (Figure 5A); compounds **ag** and **an** inhibited spheroid growth to the same extent as compound **aa**, and compound **au** exhibited significantly lesser growth inhibitory effects than compound **aa** (Figure 5A). Importantly, these data also reveal that Complex I inhibitors which exhibit limited antiproliferative effects on cancer cells cultured in 2D (see Figure 4E and Supplementary Figure S3A) may reveal a strong growth inhibitory potential in 3D models. These observations confirm a major role of the intricate exchange of metabolites between cancer cells to evaluate the anticancer potential of drugs targeting mitochondrial activity. Our data also suggest that small changes in the chemical structures of compounds exhibiting similar in vitro activity may account for significant differences when exposed to the tumor microenvironment, leading to distinct abilities to diffuse inside 3D spheroids and to cross plasma and mitochondrial membranes.

We also took advantage of 3D tumor spheroids to evaluate whether they could radiosensitize tumors upon O_2_ sparing resulting from inhibition of O_2_ consumption. When a 6 Gy irradiation was administered at day 4, a further significant reduction in spheroid growth was obtained for each tested compound (vs. treatment with each compound alone (*p* < 0.01)) (Figure 5B). Interestingly, compounds **aj** and **am** showed a net reduction in the spheroid size (i.e., not merely a decreased growth rate), indicating a significant contribution of cell death to the reduced growth (Figure 5B).

**Figure 6:**
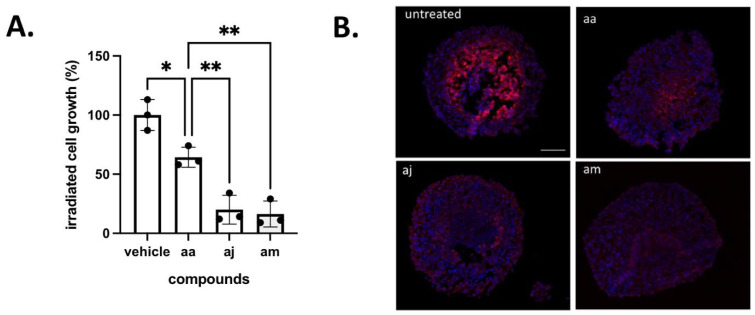
Radiosensitizing effects of selected complex I inhibitors. (**A**) Extent of surviving cancer cells post-exposure to radiotherapy and the indicated compounds (n = 3, * *p* < 0.05, ** *p* < 0.01 vs. vehicle condition). (**B**) Representative pictures of pimonidazole immunostaining revealing a reduction in the hypoxic fraction of 3D tumor CT26 spheroids induced by the indicated compound; this experiment was repeated twice with similar results.

To further prove the radiosensitizing effects, spheroids treated with compounds **aa**, **aj** and **am** were dissociated and collected cells were plated to grow for 14 days. This assay revealed that cancer cell exposed to both irradiation and compounds **aj** and **am** exhibited a much lesser ability to re-grow, suggesting that the extent of catastrophic mitosis was significantly higher than with reference compound **aa** (Figure 6A). Finally, to document the expected re-oxygenation, spheroids treated with compounds **aa**, **aj** and **am** were exposed to the hypoxia probe pimonidazole. While pimonidazole labelled the core of untreated spheroids, immunofluorescence staining confirmed that **aj** and **am** compounds reduced hypoxia to a larger extent than compound **aa** (Figure 6B).

## 4. Discussion

The main finding of this study is the feasibility to screen in 96-well plate format for inhibitors of mitochondrial complex I by tracking the capacity of compounds to promote L-lactate release and to further probe their ability to reach their targets in 3D-organized cancer cells. We also provide evidence that the latter 3D spheroid assay has the potential to reveal direct cytotoxic and radiosensitizing effects of OXPHOSi that remain unnoticed when using conventional 2D cancer cell cultures.

Classical cytotoxic assays cannot be used to identify OXPHOSi including complex I inhibitors from a classical in vitro screening campaign since the metabolic plasticity of cancer cells offers them the possibility to rapidly adapt to inhibition of cell respiration. The method described here aims to be used for mid- to high-throughput screening of libraries of naïve compounds. To prove the relevance of the procedure, however, we synthesized derivatives of carboxyamidotriazole, a drug reported to possibly exert OXPHOS inhibitory effects, and used our assays to optimize hit development. Remarkably, a primary assay exploring the induction of L-lactate release as a direct response to respiration inhibition led us to document that 13 out of 28 derivatives (exhibiting minimal structure alterations when compared with reference compound) were deprived of this capacity. Importantly, these 13 compounds also failed to show complex I inhibitory activity proving the validity of the procedure while 12 out of the remaining 15 inducers of L-lactate release were further validated as complex I inhibitors in our secondary assay. These results indicate the great value of the L-lactate-based pre-screening primary assay carried out in volume as low as 10µL. We used here a semi-automated approach but a robotic plate handler could easily convert this simplistic assay in a fully automated strategy. It should also be emphasized that the same equipment can measure glucose content in the extracellular medium. Glucose concentration evolves as mirror of lactate concentration upon stimulation of glycolysis so that measurements of the two parameters could offer a yet more robust primary assay. Simultaneous measurements of glucose consumption could also discriminate OXPHOSi from cytotoxic drugs which may increase extracellular lactate concentration without consumption of glucose (because of the release of internal lactate pool upon loss of plasma membrane integrity).

While induction of L-lactate release can be used to restrict a list of hits endowed with OXPHOS inhibitory activity, the nature of the drug-induced mitochondrial defects may vary. We recently reported that the blockade of the mitochondrial transport of pyruvate led to a progressive shift toward glycolysis and the associated increase in L-lactate release [15]. Inhibiting the activity of TCA cycle enzymes could also reduce mitochondrial activity without directly impacting OXPHOS. In the current study, the reversibility of the proposed complex I inhibition was examined by the consecutive addition of succinate to fuel complex II. Although supercomplex assembly supports structural interdependency between mitochondrial complexes [41,42,43], the succinate-dependent rescue of OCR inhibition (see Figure 4A–D and Supplementary Figure S2) represents strong evidence to claim that selected hits represent *bona fide* complex I inhibitors. Still, we cannot fully exclude a contribution of an impairment of either the mitochondrial L-lactate uptake and/or oxidation, or the availability of complex I respiratory substrates used in this study. More investigation is warranted to explore these non-exclusive options at the light of recent findings related to mitochondrial carriers [17].

Our last assay evaluating the efficacy of compounds identified as complex I inhibitors on 3D tumor spheroids proved the interest of 3D structures to further select hits for future developments. First, this assay showed that compounds with limited activity on cancer cell monolayers may exert cytostatic and even cytotoxic effects in 3D. Second, spheroid models led us to uncover a limited growth inhibitory effects for compounds **ag** and **au** despite their identification in the top 3 compounds based on the induction of L-lactate release and the inhibition of complex I. By contrast, compound **am** identified as a complex I inhibitor with an intermediary potency showed a strong inhibitory activity on spheroid growth. The compound **aj** was actually the only compound exhibiting the highest activity in the three assays of our protocol. The above conclusions actually fit our other findings related to the radio-sensitizing potential of compounds **am** and **aj** which showed the more profound inhibition of cancer cell regrowth post-irradiation supported by a large reduction in hypoxic fractions. Of note, this study mainly focused on a screening procedure to identify complex I inhibitors and even though we synthesized a variety of compounds, establishing an elaborated SAR was beyond the scope of this study. To guide future work, a scheme of the different moiety chemical modifications is presented and potential advantages of **aj** and **am** compounds are briefly discussed as Supplementary Figure S4.

## 5. Conclusions

This study documents how 96-well plate format may be used to screen for OXPHOS inhibitors and possibly identify complex I inhibitors, taking advantage of their capacity to stimulate L-lactate release while leaving OCR unaltered when using succinate as a complex II fuel. More work is warranted to exclude effects on carrier-mediated transport of metabolites which are oxidized in the respiratory chain. We also provide evidence that 3D tumor spheroid models can reveal a higher cytotoxic potential of these compounds than when it is evaluated using conventional 2D cancer cell cultures. Addiction to some metabolic paths may indeed be imposed by the tumor microenvironment recapitulated in 3D structures and leads to more pronounced drug effects. Importantly, 3D spheroids may also help selecting drug candidates by identifying compounds more prone to reach their targets in a tumor mimicking environment, and lead to reoxygenation and associated radiosensitization.

## Figures and Tables

**Table 1:** List of **aa** compound derivatives and corresponding structures.

Compound No.	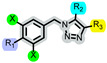 i (aa–aw)				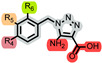 ii (ba–bf)		
	X	R_1_	R_2_	R_3_	R_4_	R_5_	R_6_
**aa**	-Cl	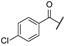	-NH_2_	-CONH_2_	-	-	-
**ab**	-Cl	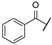	-NH_2_	-CONH_2_	-	-	-
**ac**	-Cl	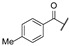	-NH_2_	-CONH_2_	-	-	-
**ad**	-Cl	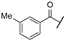	-NH_2_	-CONH_2_	-	-	-
**ae**	-Cl	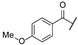	-NH_2_	-CONH_2_	-	-	-
**af**	-Cl	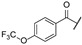	-NH_2_	-CONH_2_	-	-	-
**ag**	-Cl	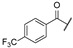	-NH_2_	-CONH_2_	-	-	-
**ah**	-Cl	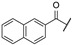	-NH_2_	-CONH_2_	-	-	-
**ai**	-Cl	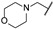	-NH_2_	-CONH_2_	-	-	-
**aj**	-Cl	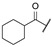	-NH_2_	-CONH_2_	-	-	-
**ak**	-Cl	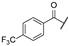	-NH_2_	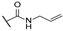	-	-	-
**al**	-Cl	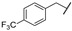	-NH_2_	-CONH_2_	-	-	-
**am**	-Cl	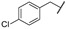	-NH_2_	-CONH_2_	-	-	-
**an**	-F	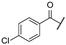	-NH_2_	-CONH_2_	-	-	-
**ao**	-Cl	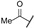	-NH_2_	-CONH_2_	-	-	-
**ap**	-Me	-H	-NH_2_	-CONH_2_	-	-	-
**aq**	-Cl	-H	-NH_2_	-CONH_2_	-	-	-
**ar**	-Cl	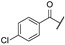	-H	-COOH	-	-	-
**as**	-Cl	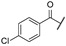	-H	-CONH_2_	-	-	-
**at**	-Cl	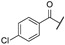	-H	-CH_2_NH_2_	-	-	-
**au**	-Cl	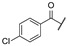	-H	-COOCH_3_	-	-	-
**av**	-Cl	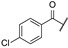	-H	-CH_2_OH	-	-	-
**aw**	-F	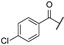	-H	-CONH_2_	-	-	-
**ba**	-	-	-	-	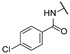	-H	-H
**bb**	-	-	-	-	-H	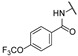	-H
**bc**	-	-	-	-	-H	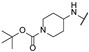	-H
**bd**	-	-	-	-	-H	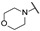	-H
**be**	-	-	-	-	-H	-H	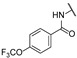
**bf**	-	-	-	-	-H	-H	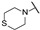

**Table 2:** Evaluation of the activity of **aa** compound derivatives in different screening assays. Assays include stimulation of L-lactate release (assay #1), mitochondrial complex I inhibition (assay #2) and 3D tumor spheroids growth inhibitory effects (assay #3).

	Assay #1	Assay #2	Assay #3
compounds	[LAC] increase (µmol·h^−1^·mg^−1^)	OCR inhib. (IC_50_, µM)	3D growth inhib. (%)
**aa**	~2	1 < x < 10	25 < x < 50
**ab**	~2	~10	n.d.
**ac**	~2	~10	n.d.
**ad**	~2	~10	n.d.
**ae**	~2	~10	n.d.
**af**	~2	~10	n.d.
**ag**	~4	1 < x < 10	25 < x < 50
**ah**	~2	~10	n.d.
**ai**	~2	>10	n.d.
**aj**	~4	1 < x < 10	>50
**ak**	~0	>10	n.d.
**al**	~0	>10	n.d.
**am**	~4	~10	>50
**an**	~4	~10	25 < x < 50
**ao**	~2	>10	n.d.
**ap**	~0	>10	n.d.
**aq**	~0	>10	n.d.
**ar**	~0	>10	n.d.
**as**	~2	>10	n.d.
**at**	~0	>10	n.d.
**au**	~4	1 < x < 10	<25
**av**	~0	>10	n.d.
**aw**	~0	>10	n.d.
**ba**	~0	>10	n.d.
**bb**	~0	>10	n.d.
**bc**	~0	>10	n.d.
**bd**	~0	>10	n.d.
**be**	~0	>10	n.d.
**bf**	~0	>10	n.d.

This Table is based on values determined in Figure 3, Figure 4 and Figure 5 and Supplementary Figure S2.

## Data Availability

The data presented in this study are available on request from the corresponding author.

## Supplementary Materials

The following are available online at https://www.mdpi.com/article/10.3390/cancers14215454/s1, Figure S1. Compound-induced L-lactate release and mitochondrial complex I inhibition in 4T1 mouse breast cancer cells. Figure S2. Mitochondrial complex I activity inhibition by **aa** derivatives. Figure S3. Growth inhibitory effects of hit compounds on human cancer cells. Figure S4. Scheme depicting the pharmacomodulation of compound **aa**.
