# Supplementary material for: Discovery of Mitochondrial Complex I Inhibitors as Anticancer and Radiosensitizer Drugs Based on Compensatory Stimulation of Lactate Release
Source: Cancers (Basel). 2022 Nov 6;14(21):5454. doi: 10.3390/cancers14215454 (PMC9658316; doi:10.3390/cancers14215454)
Supplement: Supplementary file 1 [file cancers-14-05454-s001.zip › cancers-1955736-supplementary.pdf]

Supplementary Figures and Information.

Supplementary Figure S1.

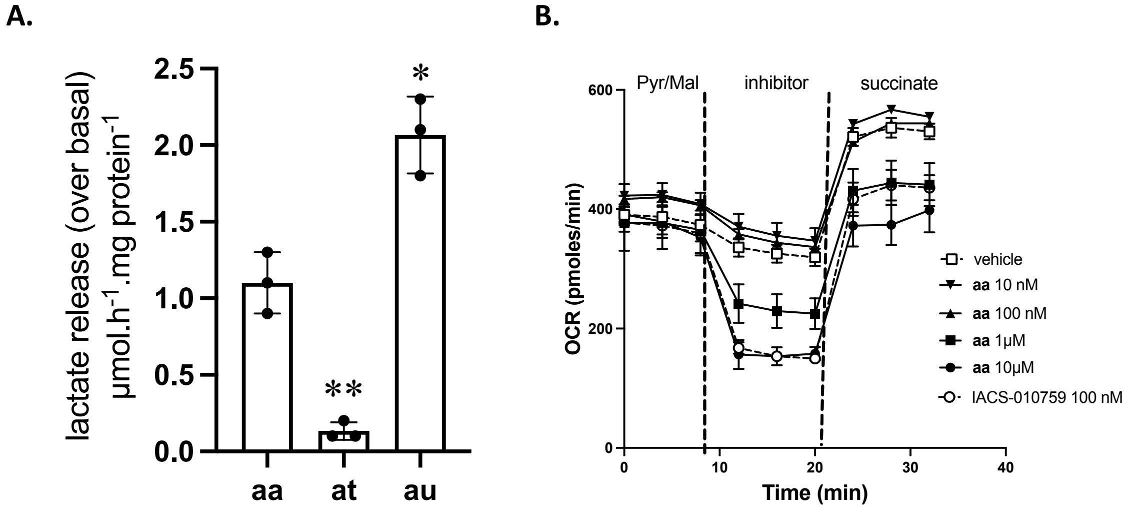

Suppl. Figure S1. A. Compound-induced lactate release in 4T1 breast cancer cells (Assay #1). Culture medium was collected after 24h exposure to the indicated compounds and lactate content was determined in a semi-automated manner. Bar graph depicts the extent of lactate secretion above the basal release as determined in the absence of any compound (n = 3, *P<0.05, **P<0.01 for lactate release *vs.* reference compound aa). **B.** Mitochondrial complex I activity inhibition in 4T1 breast cancer cells (Assay #2). Graph depicts changes in O_2 consumption rate (OCR) measured with the Seahorse technology on permeabilized 4T1 breast cancer cells upon successive addition of pyruvate/malate, compound aa and succinate. OXPHOS inhibitor IACS was used as a control (n=6 per compound).

Supplementary Figure S2.

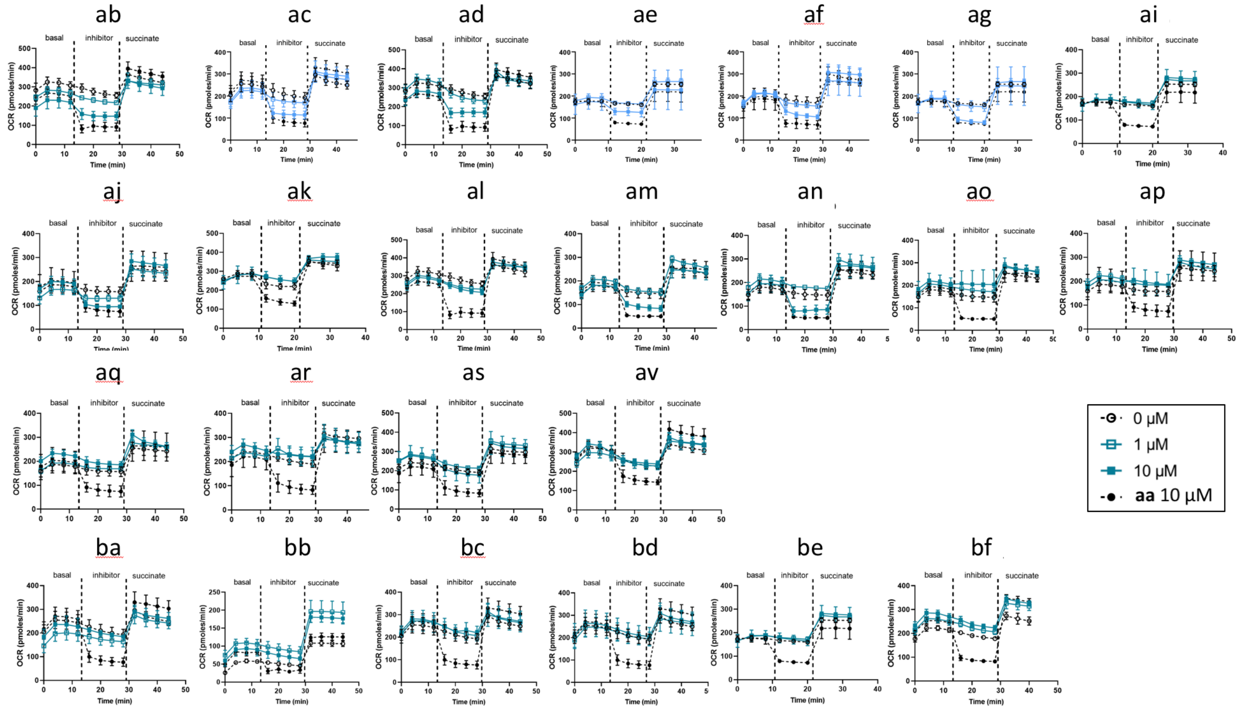

Suppl. Figure S2. Mitochondrial complex I activity inhibition (Assay #2). Graphs depict changes in O2 consumption rate (OCR) measured with the Seahorse technology on permeabilized CT26 cancer cells upon successive addition of pyruvate/malate, the indicated inhibitor and succinate. OXPHOS inhibitor IACS was used as a control in the experiments testing the indicated compounds at 1 and 10 μM (n = 6 per compound).

Supplementary Figure S3.

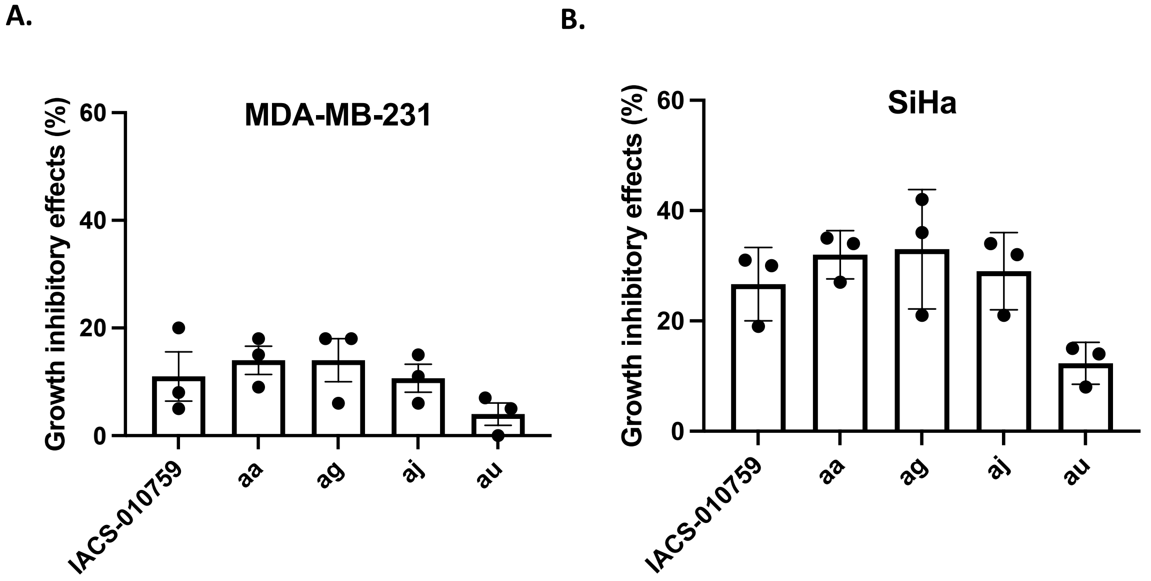

Suppl. Figure S3. Growth inhibitory effects of hit compounds on human cancer cells. MDA-MB-231 (A) and Siha (B) cancer cells were exposed to 100 nM IACS or 1 µM aa, ag, aj and au compounds; data represent the reduced extent (expressed as %) of cell viability (determined with Presto Blue) when compared with vehicle conditions (n=3).

Supplementary Figure S4.

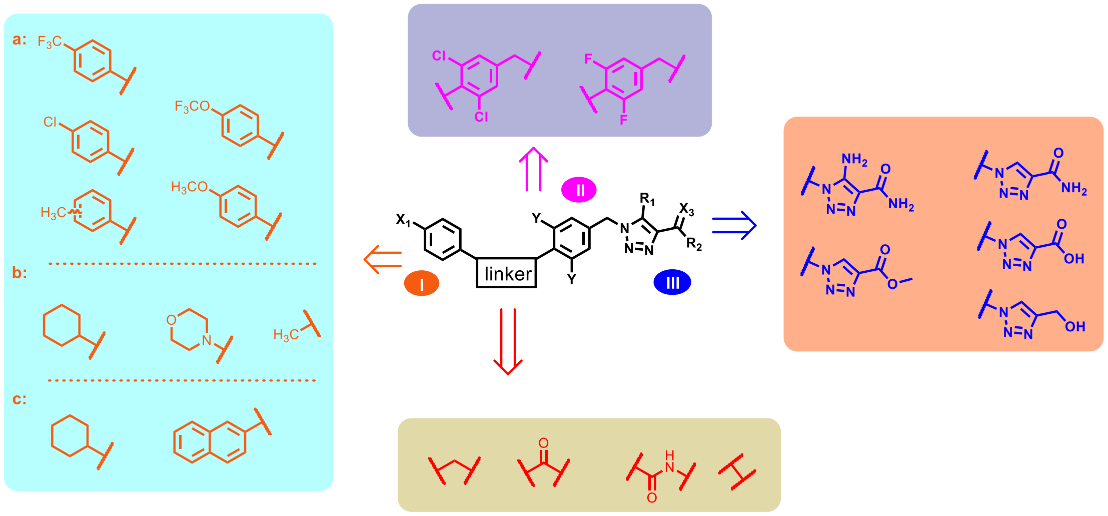

Suppl. Figure S4. Scheme depicting the pharmacomodulation of compound aa. Main chemical modifications in different moieties are summarized. First insights on SAR study based on the two most active compounds **aj** and **am** are the following. In the moiety I, we used 4-substituted phenyl group instead of 4-chlorophenyl group and the results showed that the hydrophobic substituent was more active than the hydrophilic substituent. We then used cycloalkanes instead of benzene rings to synthesize compound **aj**, which was found to be more active. In the moiety II, we investigated the effect of different linkers on the activity of the compounds. It was found that the compound **am** obtained by replacing the carbonyl group (-C=O-) with a methylene group (-CH_2-) exhibited higher activity. Altogether, hydrophobicity in moieties I and II seems to be beneficial to improve the activity.

Supplementary Information.

1. General:

Unless otherwise mentioned, reagents were purchased from commercial sources (Sigma Aldrich, TCI, Acros, FluoroChem) and used without further purification. All solvents were dried from Na or CaH_2 and purified by distillation before being used. NMR spectra were recorded at room temperature on a Bruker Avance UltraShield instrument operating at a frequency of 300 MHz for ^1H and 75 MHz for ^{13}C. Chemical shifts (δ) are reported in ppm relative to $CDCl_3$ (δ = 7.26 ppm) and DMSO-d_6 (δ = 2.50 ppm) for ^1H NMR and $CDCl_3$ (δ = 77.2 ppm) and DMSO-d_6 (δ = 39.52 ppm) for ^{13}C NMR multiplicity (s = singlet, d = doublet, t = triplet, m = multiplet, q = quartet, br = broad). Column chromatography was performed over ROCC Silica gel 60 (40-63 μm). Thin layer chromatography was performed on prepared thin layers precoated plates: Silica gel Merck 60 F254. The visualization of spots on TLC plates was done by UV light (254nm or 365 nm) or $KMnO_4$ solution staining. Mass spectra were recorded using an orbitrap Q Exactive Thermo Fisher spectrometer, which is a hybrid quadrupole-orbitrap mass spectrometer.

2. Substrates Preparation

2.1. General procedure for preparation of benzene azidomethyl

2.1.1. Preparation of (4-(azidomethyl)-2,6-dichlorophenyl)(phenyl)methanone

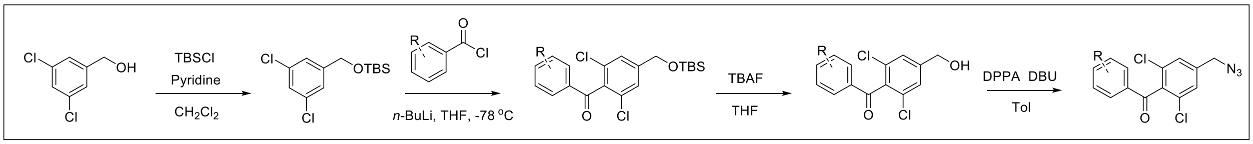

The compound (3,5-dichlorophenyl) methanol (5.0 g, 28.24 mmol) in dichloromethane (30 ml) solution of *tert*-butyidimethylsilyl chloride (5.4 ml, 31.07 mmol), pyridine (2.7 ml, 33.89 mmol) was added under argon at 0℃. After stirring overnight at room temperature and after completion of the reaction, the mixture was washed with HCl aq (1M) and brine by extraction with CH_2Cl_2, dried over anhydrous sodium sulfate, filtered and concentrated under reduced pressure to give a colorless oil. The residue was purified by silica gel column chromatography (petroleum ether/ ethyl acetate 100/1) to give the compound *tert*-butyl ((3,5-dichlorobenzyl) oxy) dimethylsilane as a colorless oil (7.5 g, 89.0%). **^{1}H-NMR** (300MHz, $CDCl_3$): δ 7.23-7.19 (m, 3H), 4.68 (s, 2H), 0.91 (s, 9H), 0.11(s, 6H).

To a solution of *tert*-butyl ((3,5-dichlorobenzyl)oxy) dimethylsilane (1.0 g, 3.43 mmol) was dissolved in anhydrous THF (7.0 mL) followed the addition of *n*-BuLi (2.5 M solution in hexane, 1.4 mL, 3.4 mmol) dropwise at -78 ℃. The resulting solution was stirred at -78 ℃ for 30 min followed by the addition of 4-chlorobenzoyl

chloride (460 µL, 3.43 mmol). The reaction was stirred at -78 ℃ to room temperature for 3 h then quenched with HCl aq (1M). The extractions were performed with ethyl acetate and the combined organic layers were dried over $MgSO_4$, filtered and concentrated under reduced pressure to give a pale-yellow oil. The crude product was used for next step without further purification. The crude product in THF (5 mL) was added TBAF (1.0 M solution in THF, 8.6 mL, 8.58 mmol) at room temperature and stirred at room temperature for 3 h then quenched with aqueous saturated NH_4Cl solution. The extractions were performed with ethyl acetate and the combined organic layers were dried over $MgSO_4$ followed by concentrated under reduced pressure to give a brown powder. The residue was purified by silica gel column chromatography (CH_2Cl_2/ MeOH 50/1-30/1) to give the compound (4-chlorophenyl)(2,6-dichloro-4-(hydroxymethyl)phenyl) methanone as an off-white solid (776 mg, 70 %).**^{1}H-NMR** (300MHz, DMSO-*d_6*): δ 7.77 (d, *J*= 6.0 Hz, 2H), 7.67 (d, *J*= 6.0 Hz, 2H), 7.56 (s, 2H), 5.57 (t, *J*= 6.0 Hz, 1H), 4.60 (d, *J*= 6.0 Hz, 2H). **^{13}C-NMR** (75MHz, DMSO-*d_6*): δ191.60, 148.36, 140.42, 134.84, 133.99, 131.46, 130.78, 130.23, 126.46, 61.83.

To a solution of (4-chlorophenyl)(2,6-dichloro-4-(hydroxymethyl)phenyl) methanone (200 mg, 0.63 mmol) dissolved in THF 8.0 mL was treated dropwise 1,8-diazabicyclo[5.4.0]undec-7-ene DBU (115 µL, 0.76 mmol), after 5 min added the diphenylphosphoryl azide DPPA (206 µL, 0.95 mmol) at room temperature. Stirring the mixture for overnight at the same temperature, the mixture was added aqueous saturated ammonium chloride. The mixture was extracted with ethyl acetate, and the combined organic extract was washed with brine, dried over $MgSO_4$ followed by concentrated under reduced pressure. The residue was purified by silica gel column chromatography (petroleum ether/ ethyl acetate 30/1-10/1) to give the compound (4-(azidomethyl)-2,6-dichlorophenyl)(4-chlorophenyl)methanone as a light yellow oil (162 mg, 75 %). **^{1}H-NMR** (300MHz, $CDCl_3$): δ 7.76 (d, *J*= 9.0 Hz, 2H), 7.69 (d, *J*= 9.0 Hz, 2H), 7.56 (s, 2H), 4.60 (s, 2H). **^{13}C-NMR** (75MHz, $CDCl_3$): δ 191.02, 141.09, 139.50, 136.86, 133.72, 132.36, 130.93, 129.45, 127.38, 53.26.

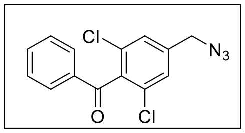

According to the method of (4-(azidomethyl)-2,6-dichlorophenyl)(4-chlorophenyl)methanone, to give the compound (4-(azidomethyl)-2,6-dichlorophenyl)(phenyl)methanone (139 mg, 64 %) as a yellow oil. **^{1}H-NMR** (300MHz, $CDCl_3$): δ 7.84-7.81 (m, 2H), 7.76-7.62 (m, 1H) 7.52-7.47 (m, 2H), 7.36 (s, 2H), 4.43(s, 2H).

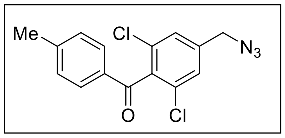

According to the method of (4-(azidomethyl)-2,6-dichlorophenyl)(4-chlorophenyl)methanone, to give the

compound (4-(azidomethyl)-2,6-dichlorophenyl)(*p*-tolyl)methanone (183 mg, 84 %) as a yellow oil. **^{1}H-NMR** (300MHz, $CDCl_3$): δ 7.68 (d, *J*= 9.0 Hz, 2H), 7.32 (s, 2H), 7.24 (d, *J*= 9.0 Hz, 2H), 4.39 (s, 2H), 2.40 (s, 3H). **^{13}C-NMR** (75MHz, $CDCl_3$): δ 191.82, 145.67, 139.08, 137.60, 132.94, 132.36, 130.08, 129.77, 127.31, 126.15, 120.27, 120.20, 53.29, 21.89.

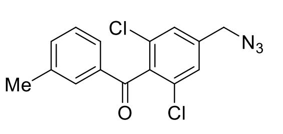

According to the method of (4-(azidomethyl)-2,6-dichlorophenyl)(4-chlorophenyl) methanone, to give the compound(4-(azidomethyl)-2,6-dichlorophenyl)(*m*-tolyl) methanone (174 mg, 80 %) as a yellow oil. **^{1}H-NMR** (300MHz, $CDCl_3$): δ 7.68 (s, 1H), 7.55 (d, *J*= 9.0 Hz, 1H), 7.44 (d, *J*= 9.0 Hz, 1H), 7.39-7.34 (m, 3H) 4.43 (s, 2H), 2.41 (s, 3H).

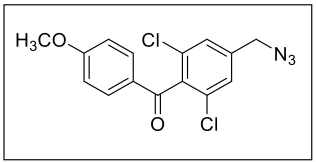

According to the method of (4-(azidomethyl)-2,6-dichlorophenyl)(4-chlorophenyl)methanone, to give the compound (4-(azidomethyl)-2,6-dichlorophenyl)(4-methoxyphenyl)methanone (188 mg, 87 %) as a colorless oil. **^{1}H-NMR** (300MHz, $CDCl_3$): δ 7.80 (d, *J*= 9.0 Hz, 2H), 7.35 (s, 2H), 6.97 (d, *J*= 9.0 Hz, 2H), 4.42 (s, 2H), 3.87 (s, 3H). **^{13}C-NMR** (75MHz, $CDCl_3$): δ 190.64, 164.64, 139.04, 137.61, 132.31, 132.05, 128.45, 127.30, 114.32, 55.59, 53.25.

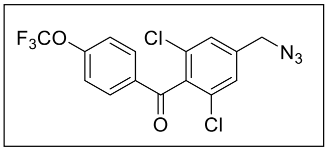

According to the method of (4-(azidomethyl)-2,6-dichlorophenyl)(4-chlorophenyl)methanone, to give the compound (4-(azidomethyl)-2,6-dichlorophenyl)(4-(trifluoromethyloxy)phenyl)methanone (142 mg, 66 %) as a yellow oil. **^{1}H-NMR** (300MHz, $CDCl_3$): δ 7.89 (d, *J*= 9.0 Hz, 2H), 7.37 (s, 2H), 7.32 (d, *J*= 9.0 Hz, 2H), 4.44 (s, 2H). **^{13}C-NMR** (75MHz, $CDCl_3$): δ 190.70, 153.54, 139.57, 136.79, 133.44, 132.37, 131.67, 130.08, 127.39, 120.59, 53.25.

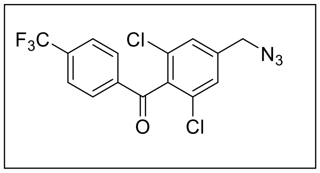

According to the method of (4-(azidomethyl)-2,6-dichlorophenyl)(4-chlorophenyl)methanone, to give the compound (4-(azidomethyl)-2,6-dichlorophenyl)(4-(trifluoromethyl)phenyl)methanone (144 mg, 67 %) as a yellow oil. **^{1}H-NMR** (300MHz, $CDCl_3$): δ 7.94 (d, J= 9.0 Hz, 2H), 7.76 (d, J= 9.0 Hz, 2H), 7.39 (s, 2H), 4.45 (s, 2H).

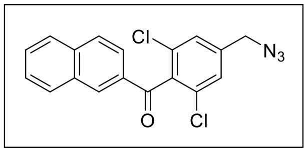

According to the method of (4-(azidomethyl)-2,6-dichlorophenyl)(4-chlorophenyl)methanone, to give the compound (4-(azidomethyl)-2,6-dichlorophenyl)(naphthalen-2-yl)methanone (161 mg, 75 %) as a yellow oil. **^{1}H-NMR** (300MHz, $CDCl_3$): δ 8.17 (s, 1H), 8.04-7.89 (m, 3H), 7.66-7.61 (m, 1H), 7.39 (s, 2H), 7.28-7.25 (m, 1H), 4.47 (s, 2H).

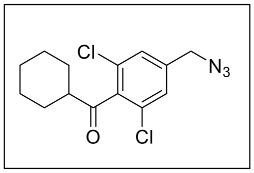

According to the method of (4-(azidomethyl)-2,6-dichlorophenyl)(4-chlorophenyl)methanone, to give the compound (4-(azidomethyl)-2,6-dichlorophenyl)(cyclohexyl)methanone (144 mg, 66 %) as a yellow oil. **^{1}H-NMR** (300MHz, $CDCl_3$): δ 7.27 (s, 2H), 4.32 (s, 2H), 2.34-2.29 (m, 1H), 1.96-1.90 (m, 2H), 1.76-1.52 (m, 5H), 1.47-1.16 (m, 3H).

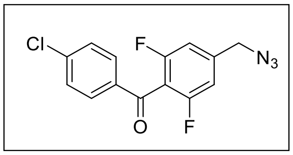

According to the method of (4-(azidomethyl)-2,6-dichlorophenyl)(4-chlorophenyl)methanone, to give the compound (4-(azidomethyl)-2,6-difluorophenyl)(4-chlorophenyl)methanone (176 mg, 81 %) as a yellow oil. **^{1}H-NMR** (300MHz, $CDCl_3$): δ 7.79 (dd, J= 3.0, 6.0 Hz, 2H), 7.47 (dd, J= 3.0, 6.0 Hz, 2H), 7.00 (m, 2H), 4.45(s, 2H).

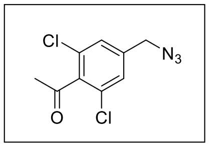

According to the method of (4-(azidomethyl)-2,6-dichlorophenyl)(4-chlorophenyl)methanone, to give the compound 1-(4-(azidomethyl)-2,6-dichlorophenyl)ethan-1-one (189 mg, 85 %) as a yellow oil. **^{1}H-NMR** (300MHz, $CDCl_3$): δ 7.28 (s, 2H), 4.36 (s, 2H), 2.57(s, 3H).

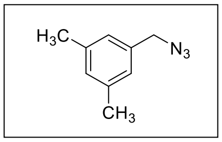

According to the method of (4-(azidomethyl)-2,6-dichlorophenyl)(4-chlorophenyl)methanone, to give the compound 1-(azidomethyl)-3,5-dimethylbenzene (191 mg, 81 %) as a colorless oil. **^{1}H-NMR** (300MHz, $CDCl_3$): δ 6.98 (s, 1H), 6.93 (s, 2H), 4.26 (s, 2H), 2.33(s, 6H).

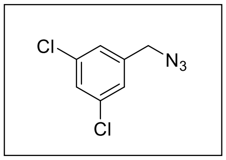

According to the method of (4-(azidomethyl)-2,6-dichlorophenyl)(4-chlorophenyl)methanone, to give the compound 1-(azidomethyl)-3,5-dichlorobenzene (183 mg, 80 %) as a colerless oil. **^{1}H-NMR** (300MHz, $CDCl_3$): δ 7.35 (s, 2H), 7.25 (s, 1H), 4.33 (s, 2H).

2.1.2. Preparation of 5-(azidomethyl)-1,3-dichloro-2-(4-chlorobenzyl)benzene

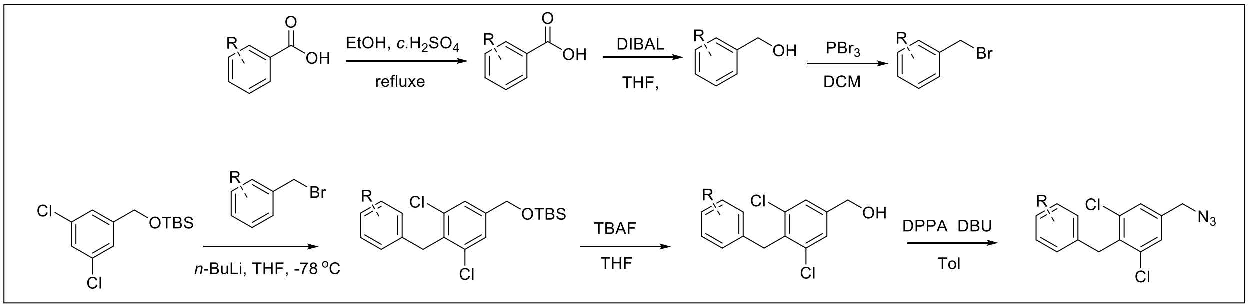

To a solution of 4-chlorobenzoic acid (1.0 g, 3.39 mmol) dissolved in EtOH 15 mL was treated dropwise concentrated sulfuric acid (90 μL, 1.60 mmol), the mixture was heated at reflux for overnight. The mixture was cooled, concentrated under reduced pressure and diluted with water. The aqueous layer was extracted with ethyl acetate, and the combined organic extract was washed with brine, dried over $MgSO_4$ followed by concentrated

under reduced pressure to afford crude ethyl 4-chlorobenzoate as a yellow solid. The crude compound was not purified to the next step. To a solution of ethyl 4-chlorobenzoate dissolved in THF 15 mL was cooled to 0 ^oC. The diisobutylaluminium hydride (1M solution in THF, 12.8 ml, 12.78 mmol) was added to dropwise at 0 ^oC, it was them stirred at 0 ^oC 2h which is was quenched with 10% Rochelle's salt solution. The mixture was then stirred for more 1h until the organic layer separates out. The mixture was extracted with ethyl acetate, and the combined organic extract was washed with brine, dried over $MgSO_4$ followed by concentrated under reduced pressure. The residue was purified by silica gel column chromatography (petroleum ether/ ethyl acetate 5/1-3/1) to give the compound (4-chlorophenyl)methanol as a light yellow soild (675 mg, 74 %). ^{1}**H-NMR** (300MHz, $CDCl_3$): δ 7.50-7.20 (m, 4H), 4.45 (s, 2H), 2.80 (s, 1H).

To a solution of (4-chlorophenyl)methanol (500 mg, 3.51 mmol) in dry CH_2Cl_2 7 mL was slowly added phosphorus tribromide (335 μL, 3.51 mmol) at 0 °C, and the mixture was stirred at room temperature for 3 h. The mixture was cooled at 0 °C, and quenched with water. The mixture was extracted with ethyl acetate and washed with water, before dried over $MgSO_4$. The residue was purified by silica gel column chromatography (petroleum ether/ ethyl acetate 50/1) to give the compound 4-chlorobenzyl bromide as a colorless soild (516 mg, 72 %). ^{1}**H-NMR** (300MHz, $CDCl_3$): δ 7.50-7.20 (m, 4H), 4.46 (s, 2H).

The next step according to the method of (4-(azidomethyl)-2,6-dichlorophenyl)(4-chlorophenyl)methanone to give the below compound.

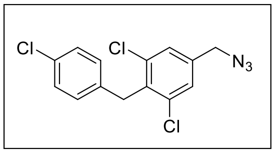

5-(azidomethyl)-1,3-dichloro-2-(4-chlorobenzyl)benzene (153 mg, 71 %) as a yellow oil. ^{1}**H-NMR** (300MHz, $CDCl_3$): δ 7.59 (s, 2H), 7.43 (d, J= 9.0 Hz, 2H), 7.29 (d, J= 9.0 Hz, 2H), 4.56 (s, 2H), 4.51 (s, 2H).

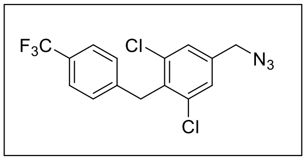

5-(azidomethyl)-1,3-dichloro-2-(4-(trifluoromethyl)benzyl)benzene (149 mg, 69 %) as a yellow oil. ^{1}**H-NMR** (300MHz, $CDCl_3$): δ 7.53 (d, J= 9.0 Hz, 2H), 7.34-7.27 (m, 4H), 4.38 (s, 2H), 4.34 (s, 2H).

2.1.3. Preparation of 4-(4-(azidomethyl)-2,6-dichlorobenzyl)morpholine

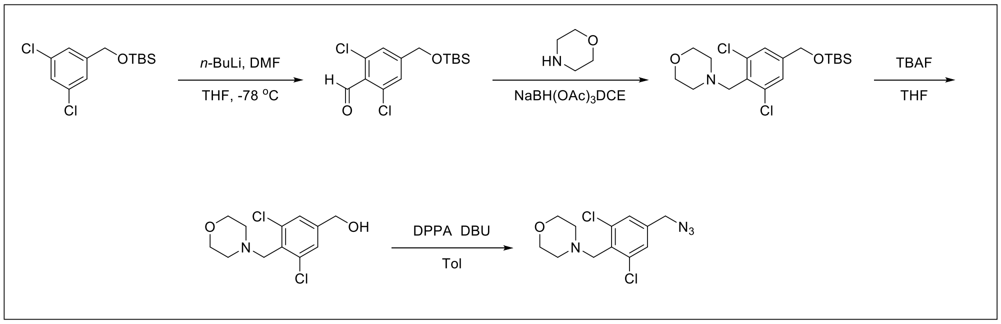

To a solution of *tert*-butyl((3,5-dichlorobenzyl)oxy)dimethylsilane (2.0 g, 6.87 mmol) in anhydrous THF at -78 °C, under Ar atmosphere, was added dropwise *n*-BuLi (2.9 mL of a 2.50 M solution in hexane, 7.21 mmol). After 40 min, anhydrous DMF (640 µL, 8.24 mmol) was added slowly dropwise to the solution. After the addition, the solution was stirred at -78 °C for 2 h then quenched with 1M HCl. The extractions were performed with ethyl acetate and the combined organic layers was washed with water, brine, dried with $MgSO_4$ and filtered. The solvent was removed under reduced pressure to give residue product, the residue was purified by silica gel column chromatography (petroleum ether/ ethyl acetate 50/1) to give the compound 4-(((*tert*-butyldimethylsilyl)oxy)methyl)-2,6-dichlorobenzaldehyde, as a colorless solid (1.8 g, 82 %). **^{1}H-NMR** (300MHz, DMSO-d_6): δ 10.34 (s, 1H), 7.48 (s, 2H), 4.79 (s, 2H), 0.91 (s, 9H), 0.10 (s, 6H).

Preparation $NaBH(OAc)_3$ triacetoxyborohydride

To a solution of sodium borohydride (590 mg, 15.66 mmol) in the anhydrous dichloroethane the temperature was kept between 15 and 20 °C, then added the dropwise glacial acetic acid (2.9 g, 50.11 mmol). After addition of acetic acid was completed, the mixture was warmed to ambient temperature and stirred for 3-4 h.

To a solution of 4-(((*tert*-butyldimethylsilyl)oxy)methyl)-2,6-dichlorobenzaldehyde (1.0 g, 3.13 mmol) and the morpholine (1.4 ml, 15.66 mmol) in the dichloroethane 10 ml was added dropwise in the triacetoxyborohydride (above solution), and the mixture was stirred and allowed to warm room temperature 24 h under Argon. The solution was quenched by added aqueous saturated $NaHCO_3$, the extractions were performed with CH_2Cl_2 and the combined organic layers was washed with water, brine, dried with $MgSO_4$ and filtered. The solvent was removed under reduced pressure to give the crude compound. The crude compound was not purified to the next step. The crude 4-(4-(((*tert*-butyldimethylsilyl)oxy)methyl) -2,6- dichlorobenzyl) morpholine in THF (8 mL) was added TBAF (1.0 M solution in THF, 7.7 mL, 7.68 mmol) at room temperature and stirred at room temperature for 4 h then quenched with aqueous saturated NH_4Cl solution. The extractions were performed with ethyl acetate and the combined organic layers were dried over $MgSO_4$ followed by concentrated under reduced pressure to give a brown solid. The residue was purified by silica gel column chromatography (CH_2Cl_2/ MeOH 50/1-40/1) to give the compound (3,5-dichloro-4- (morpholinomethyl) phenyl) methanol as a light yellow solid (610 mg, 71 %). **^{1}H-NMR** (300MHz, $CDCl_3$): δ 7.31 (s, 2H), 5.30 (s, 1H), 4.65 (s, 2H), 3.73 (s, 2H), 3.64 (dd, J= 9.0 Hz,

3.0 Hz, 4H), 2.57 (dd, *J*= 9.0 Hz, 3.0 Hz, 4H). **^{13}C-NMR** (75MHz, $CDCl_3$): δ 140.69, 135.39, 131.25, 124.81, 65.46, 62.02, 55.00, 51.80.

To a solution of (3,5-dichloro-4-(morpholinomethyl)phenyl)methanol (500 mg, 1.81 mmol) dissolved in THF 8.0 mL was treated dropwise 1,8-diazabicyclo[5.4.0]undec-7-ene DBU (340 μL, 2.26 mmol), after 10 min added the diphenylphosphoryl azide DPPA (590 μL, 2.72 mmol) at room temperature. Stirring the mixture for overnight at the same temperature, the mixture was added aqueous saturated ammonium chloride. The mixture was extracted with ethyl acetate, and the combined organic extract was washed with brine, dried over $MgSO_4$ followed by concentrated under reduced pressure. The residue was purified by silica gel column chromatography (petroleum ether/ ethyl acetate 30/1-10/1) to give the compound (4-(azidomethyl) -2,6-dichlorophenyl) (4-(trifluoromethyl)phenyl)methanone as a light yellow oil (431 mg, 79 %).**^{1}H-NMR** (300MHz, $CDCl_3$): δ 7.27 (s, 2H), 4.31 (s, 2H), 3.74 (s, 2H), 3.65 (dd, *J*= 3.0, 9.0 Hz, 4H), 2.56 (dd, *J*= 3.0, 9.0 Hz, 4H). **^{13}C-NMR** (75MHz, $CDCl_3$): δ 140.69, 135.39, 131.25, 124.81, 65.46, 62.02, 55.00, 51.80.

2.1.4. Preparation of *N*-(4-(azidomethyl)phenyl)-4-(trifluoromethyl)benzamide

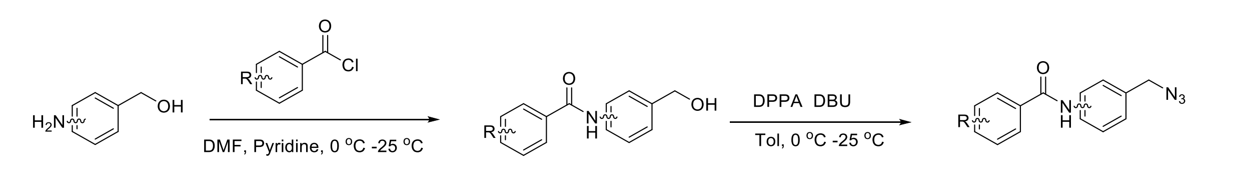

To a solution of (2-aminophenyl)methanol (1.0 g, 8.12 mmol) in the CH_2Cl_2 at 0 °C was treated dropwise with TEA (1.2 ml, 8.53 mmol) and 4-(trifluoromethoxy)benzoyl chloride (1.3 ml, 8.12 mmol). The mixture was stirred and allowed to warm room temperature 4h under Argon. The solution was quenched by adding saturation NH_4Cl (aq). The solution extractions were performed with CH_2Cl_2 and the combined organic layers was washed with water, brine, dried with $MgSO_4$ and filtered. The solvent was removed under reduced pressure to give the crude compound. The residue was purified by silica gel column chromatography (CH_2Cl_2/ MeOH 50/1) to give the compound *N*-(2-(hydroxymethyl)phenyl)-4-(trifluoromethoxy)benzamide as a yellow powder (1.78 g, 70 %). **^{1}H-NMR** (300MHz, DMSO-*d*$_6$): δ 10.17 (s, 1H), 8.06 (d, *J*= 9.0 Hz, 2H), 7.68 (dd, *J*= 1.5, 7.1 Hz, 1H), 7.55 (d, *J*= 9.0 Hz, 2H), 7.45 (dd, *J*= 1.5, 7.1 Hz, 1H), 7.30 (t, *J*= 6.0 Hz, 1H), 7.21 (t, *J*= 6.0 Hz, 1H), 5.54 (t, *J*= 6.0 Hz, 1H), 4.59 (d, *J*= 6.0 Hz, 2H).

To a solution of *N*-(2-(hydroxymethyl)phenyl)-4-(trifluoromethoxy)benzamide (200 mg, 0.64 mmol) dissolved in THF 10 mL was treated dropwise 1,8-diazabicyclo[5.4.0]undec-7-ene DBU (125 μL, 0.13 mmol), after 10 min added the diphenylphosphoryl azide DPPA (181 μL, 0.84 mmol) at room temperature. Stirring the mixture for overnight at the same temperature, the mixture was added aqueous saturated ammonium chloride. The mixture

was extracted with ethyl acetate, and the combined organic extract was washed with brine, dried over $MgSO_4$ followed by concentrated under reduced pressure. The residue was purified by silica gel column chromatography (Petroleum ether/ ethyl acetate 30/1-10/1) to give the below compound.

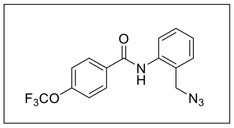

N-(2-(azidomethyl)phenyl)-4-(trifluoromethoxy)benzamide, as a yellow oil (186 mg, 86 %). **^{1}H-NMR** (300MHz, DMSO-*d6*): *δ* 10.20 (s, 1H), 8.11 (dd, *J*= 3.0, 6.0 Hz, 2H), 7.55 (dd, *J*= 3.0 Hz, 6.0 Hz, 1H), 7.47 (dd, *J*= 3.0, 6.0 Hz, 2H), 7.43-7.30 (m, 2H), 4.52 (s, 2H). **^{13}C-NMR** (75MHz, DMSO-*d6*): *δ* 164.57, 150.57, 135.97, 133.43, 131.44, 130.44, 130.12, 129.49, 50.63.

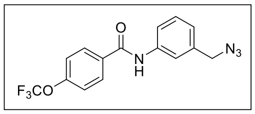

According to the method of *N*-(2-(azidomethyl)phenyl)-4-(trifluoromethoxy)benzamide to give the compound *N*-(3-(azidomethyl)phenyl)-4-(trifluoromethoxy)benzamide (167 mg, 77 %) as a yellow solid. **^{1}H-NMR** (300MHz, DMSO-*d6*): *δ* 10.20 (s, 1H), 8.10 (d, *J*= 6.0 Hz, 2H), 7.83 (s, 1H), 7.73 (d, *J*= 6.0 Hz, 1H), 7.55 (d, *J*= 6.0 Hz, 2H), 7.39 (t, *J*= 9.0, 7.1 Hz, 1H), 7.12 (t, *J*= 6.0 Hz, 1H), 4.47 (s, 2H). **^{13}C-NMR** (75MHz, DMSO-*d6*): *δ* 164.40, 150.48, 139.33, 136.18, 133.96, 130.42, 130.08, 129.05, 126.41, 123.78, 120.73, 120.11, 120.02, 53.67.

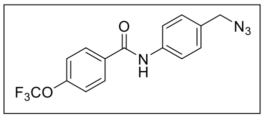

According to the method of *N*-(2-(azidomethyl)phenyl)-4-(trifluoromethoxy)benzamide to give the compound *N*-(4-(azidomethyl)phenyl)-4-(trifluoromethoxy)benzamide (139 mg, 64 %) as a yellow solid. **^{1}H-NMR** (300MHz, DMSO-*d6*): *δ* 10.43 (s, 1H), 8.08 (d, *J*= 9.0 Hz, 2H), 7.80 (d, *J*= 9.0 Hz, 2H), 7.55 (d, *J*= 9.0 Hz, 2H), 7.37 (d, *J*= 6.0 Hz, 2H), 4.41 (s, 2H). **^{13}C-NMR** (75MHz, DMSO-*d6*): *δ* 164.88, 139.37, 134.48, 131.28, 130.90, 130.55, 129.51, 121.21, 120.93, 53.73.

2.1.5. Preparation of 4-(2-(azidomethyl)phenyl)morpholine

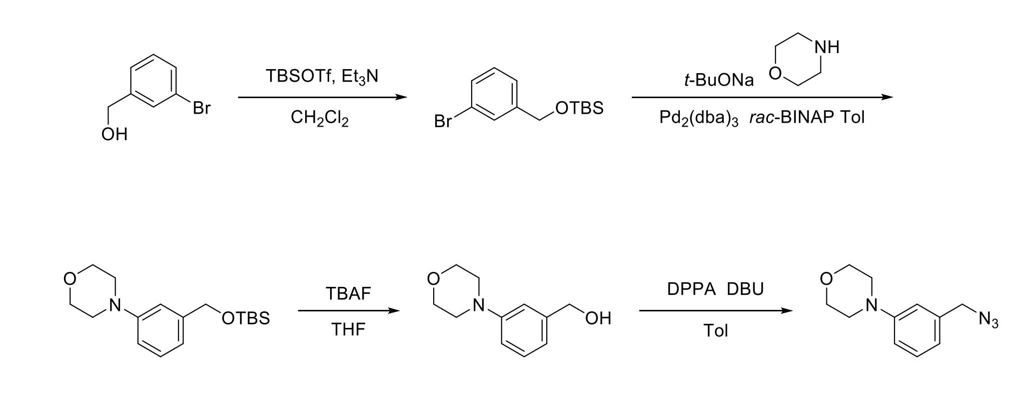

The compound (3-bromophenyl)methanol (2.0 g, 10.69 mmol) in dichloromethane (10 ml) solution of *tert*-butyldimethylsilyl trifluoromethanesulfonate TBSOTf (2.48 ml, 10.69 mmol), triethylamine (1.79 ml, 12.83 mmol) was added under argon at 0°C. After stirring 3h at room temperature and after completion of the reaction, the mixture were washed with HCl aq (1M) and brine by extraction with CH_2Cl_2, dried over anhydrous sodium sulfate, filtered and concentrated under reduced pressure to give a colorless oil. The residue was purified by silica gel column chromatography (Petroleum ether/ ethyl acetate 50/1) to give the compound ((3-bromobenzyl)oxy)(*tert*-butyl)dimethylsilane as a colorless oil (3.11 g, 97%). **^{1}H-NMR** (300MHz, DMSO-d_6): 7.49-7.48 (m, 1H), 7.46-7.42 (m, 1H), 7.32-7.30 (m, 2H), 4.71 (s, 2H), 0.90 (s, 9H), 0.08 (s, 6H).

A mixture of tris-(dibenzylideneacetone) dipalladium $Pd_2(dba)_3$ (19 mg, 0.02 mmol, 3 mol %), the ligand 2-dicyclohexylphosphino -2′,6′- dimethoxybiphenyl *rac*-BINAP (37 mg, 0.06 mmol, 9 mol %) and 10 mL of anhydrous toluene was added into an oven dried flask with argon in room temperature. Then, ((3-bromobenzyl)oxy)(*tert*-butyl)dimethylsilane (200 mg, 0.66 mmol), morpholine (325 µL, 3.32 mmol) and *t*-BuONa (83 mg, 0.86 mmol) were sequentially added to the reaction mixture. Then the reaction mixture was heated to 100 °C for 12 h. After completion of the reaction, the resulting reaction mixture was slowly brought to room temperature, and then quenched by adding water and extracted with ethyl acetate. The organic layer was dried over sodium sulphate and concentrated under reduced pressure to give the crude compound. The crude product used for next step without further purification. The crude compound in THF (8 mL) was added TBAF (1.0 M solution in THF, 2.0 mL, 1.98 mmol) at room temperature and stirred at room temperature for 4 h then quenched with aqueous saturated NH_4Cl solution. The extractions were performed with ethyl acetate and the combined organic layers were dried over $MgSO_4$ followed by concentrated under reduced pressure to give a brown solid. The residue was purified by silica gel column chromatography (CH_2Cl_2/ MeOH 50/1-30/1) to give the compound (3-morpholinophenyl)methanol as a light yellow solid (92 mg, 72 %).

To a solution of (3-morpholinophenyl)methanol (200 mg, 1.03 mmol) dissolved in THF 8.0 mL was treated dropwise 1,8-diazabicyclo[5.4.0]undec-7-ene DBU (230 µL, 1.55 mmol), after added the diphenylphosphoryl

azide DPPA (336 μL, 1.55 mmol) at room temperature. Stirring the mixture for overnight at the same temperature, the mixture was added aqueous saturated ammonium chloride. The mixture was extracted with ethyl acetate, and the combined organic extract was washed with brine, dried over $MgSO_4$ followed by concentrated under reduced pressure. The residue was purified by silica gel column chromatography (Petroleum ether/ ethyl acetate 30/1-10/1) to give the compound 4-(3-(azidomethyl)phenyl) morpholine (176 mg, 78 %) as a yellow solid. **^{1}H-NMR** (300MHz, $CDCl_3$): δ 7.30-7.22 (m, 1H), 6.94-6.91 (m, 2H), 6.81-6.78 (m, 1H), 4.36 (s, 2H), 3.74 (t, *J*=3.0 Hz, 4H), 3.12 (t, *J*=3.0 Hz, 4H). **^{13}C-NMR** (75MHz, $CDCl_3$): δ 151.76, 136.69, 129.79, 119.50, 115.48, 115.20, 66.52, 54.46, 48.73.

2.1.6. Preparation of *tert*-butyl 4-((2-(azidomethyl)phenyl)amino)piperidine-1-carboxylate

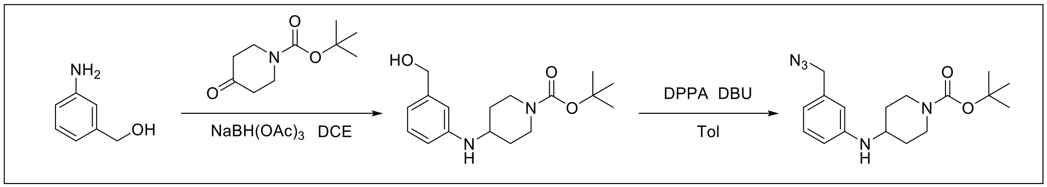

To a solution of (2-aminophenyl)methanol (1.2 g, 9.74 mmol) and the *tert*-butyl 4-oxopiperidine -1-carboxylate (2.5 g, 12.67 mmol) in the dichloroethane 10ml was added triacetoxyborohydride (6.2 g, 29.23 mmol) in the dichloroethane, and the mixture was stirred and allowed to warm room temperature 24h under Argon. The solution was quenched by added aqueous saturated $NaHCO_3$, the extractions were performed with CH_2Cl_2 and the combined organic layers was washed with water, brine, dried with $MgSO_4$ and filtered. The solvent was removed under reduced pressure to give the crude compound. The residue was purified by silica gel column chromatography (CH_2Cl_2/ MeOH 50/1) to give the compound *tert*-butyl 4-((2-(hydroxymethyl)phenyl)amino) piperidine-1-carboxylate as a yellow solid (1.2 g, 40 %). **^{1}H-NMR** (300MHz, DMSO-*d$_6$*): δ 7.11-7.04 (m, 2H), 7.64. (d, *J*=9.0 Hz, 1H), 6.54 (t, *J*=9.0 Hz, 1H), 5.12 (t, *J*=6.0 Hz, 1H), 4.91 (d, *J*=6.0 Hz, 1H), 4.40 (d, *J*=6.0 Hz, 2H), 3.86-3.82 (m, 2H), 3.53-3.45 (m, 1H), 3.02-3.29 (m, 2H), 1.95-1.86 (m, 2H), 1.40 (s, 9H), 1.31-1.24 (m, 2H).

To a solution of *tert*-butyl 4-((2-(hydroxymethyl)phenyl)amino)piperidine-1-carboxylate (0.5 g, 1.63 mmol) dissolved in THF 10.0 mL was treated dropwise 1,8-diazabicyclo[5.4.0]undec-7-ene DBU (300 μL, 2.04 mmol), after added the diphenylphosphoryl azide DPPA (425 μL, 1.96 mmol) at room temperature. Stirring the mixture for overnight at the same temperature, the mixture was added aqueous saturated ammonium chloride. The mixture was extracted with ethyl acetate, and the combined organic extract was washed with brine, dried over $MgSO_4$ followed by concentrated under reduced pressure. The residue was purified by silica gel column chromatography (Petroleum ether/ ethyl acetate 10/1) to give the compound *tert*-butyl 4-((2-(azidomethyl)phenyl)amino)piperidine-1-carboxylate (361 mg, 67 %) as a yellow oil. **^{1}H-NMR** (300MHz,

$CDCl_3$): δ 7.17 (t, J=6.0 Hz, 1H), 6.65-6.52 (m, 3H), 4.25 (s, 2H), 4.08-4.01 (m, 2H), 3.47-3.41 (m, 1H), 2.07-2.01 (m, 2H),1.47 (s, 9H), 1.37-1.30 (m, 2H).

2.2. Preparation of *N*-allyl-2-cyanoacetamide

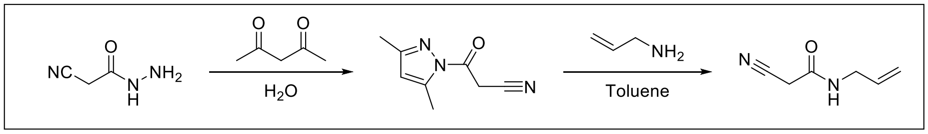

To a solution of 2-cyanoacetohydrazide (6.0 g, 60.55 mmol) in the water was added HCl (Catalytic amount) and pentane-2,4-dione (6.25 ml, 60.55 mmol). The mixture was stirred at room temperature for 2h. The precipitate was filtered, washed with water and dried to obtain a white solid as the pure product 3-(3,5-dimethyl-1*H*-pyrazol-1-yl)-3-oxopropanenitrile as a white solid (9.7 g, 98 %). **^{1}H-NMR** (300MHz, $CDCl_3$): δ 6.03 (s, 1H), 4.28 (s, 2H), 2.55 (s, 3H), 2.22 (s, 3H).**^{13}C-NMR** (75MHz, $CDCl_3$): δ 162.45, 153.88, 144.73, 113.44, 112.46, 30.95, 26.89, 14.06, 13.77.

To a solution of 3-(3,5-dimethyl-1*H*-pyrazol-1-yl)-3-oxopropanenitrile (1.0 g, 6.13 mmol) in the toluene, was added the allylamine (460 µL, 6.13 mmol). The mixture was refluxed for 30 min and then cooled room temperature. The precipitate was filtered, washed with *n*-hexane and dried to obtain a colorless solid as the pure product *N*-allyl-2-cyanoacetamide (649 mg, 85 %) as a off-white solid**.** **^{1}H-NMR** (300MHz, DMSO-d_6): δ 8.38 (s, 1H), 5.84-5.73 (m, 1H), 5.19-5.16 (m, 1H), 3.72 (t, J= 3.0 Hz, 2H), 3.65 (s, 2H). **^{13}C-NMR** (75MHz, DMSO-d_6): δ 162.39, 134.94, 116.62, 116.03, 103.62, 41.78, 25.69.

2.3. Preparation of propiolic acid amide

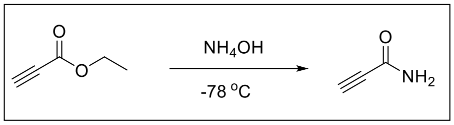

Ethyl propiolate (1ml g, 11.51 mmol) was added to the stirred aqueous ammonia (25 %, 9 ml, 57.57 mmol) at -78°C. drop wise over 5 mins. The resulting mixture was stirred under -78°C. for 1 h and was allowed to room temperature 3 h and after completion of the reaction. The reaction mixture was concentrated under *vacuo*. The residue was purified by silica gel column chromatography (Petroleum ether/ ethyl acetate 5/1 – 3/1) to give the compound propynoic acid amide as a light yellow solid (678 mg, 85 %). **^{1}H-NMR** (300MHz, DMSO-d_6): δ 8.06 (s, 1H), 7.60 (s, 1H), 4.06 (s, 1H).

3. Synthesis of CAI derivatives

3.1. Synthesis of CAI derivatives **aa-aq** and **ba-bf**

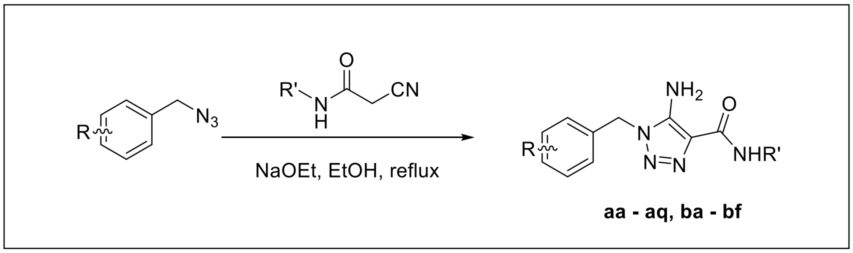

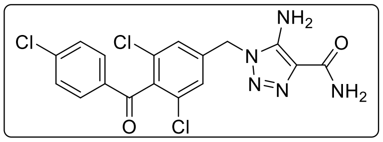

Synthesis of 5-amino-1-(3,5-dichloro-4-(4-chlorobenzoyl)benzyl)-*1H*-1,2,3-triazole-4-carboxamide (aa)

A stirred solution of sodium ethoxide (120 mg, 1.76 mmol) in EtOH (8 mL) at 0 °C was treated dropwise with 2-cyanoacetamide (148 mg, 1.76 mmol). After 10 min a solution of (4-(azidomethyl)-2,6-dichlorophenyl) (4-chlorophenyl)methanone (200 mg, 0.59 mmol) in EtOH 1 mL was added dropwise and the reaction was allowed to warm to room temperature before heating to reflux for 3 h. After cooling to room temperature the reaction was diluted by addition of water and EtOAc, the organic phase separated, the aqueous layer was extracted with EtOAc and the organic phase washed with brine, dried over anhydrous $MgSO_4$, filtered and concentrated. The residue was purified by silica gel column chromatography (CH_2Cl_2/ MeOH 50/1 to 20/1) to give the below compound 5-amino-1-(3,5-dichloro-4-(4-chlorobenzoyl)benzyl)-*1H*-1,2,3-triazole-4- carboxamide **(aa)**. (84 mg, 34 %) as a brownish yellow powder. **^{1}H-NMR** (300MHz, $CDCl_3$): δ 7.74 (dd, *J*=2.0, 7.2 Hz, 2H), 7.45 (dd, *J*=2.0, 7.2 Hz, 2H), 7.26 (s, 2H), 6.86 (s, 1H), 5.72 (s, 1H), 5.41 (s, 2H), 5.30 (s, 2H). **^{13}C-NMR** (75MHz, $CDCl_3$): δ 190.92, 164.42, 144.46, 141.33, 137.60, 137.42, 133.41, 132.84, 130.92, 129.50, 126.85, 123.05, 109.98, 50.87, 48.53. **HRMS** (APCI): calculated for $C_{17}H_{13}Cl_3N_5O_2$ $[M+H]^+$:424.0130, found: 424.0127.

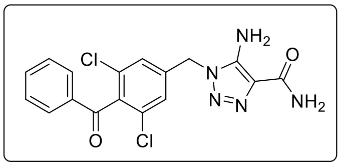

Synthesis of 5-amino-1-(4-benzoyl-3,5-dichlorobenzyl)-*1H*-1,2,3-triazole-4-carboxamide (ab)

According to the method of 5-amino-1-(3,5-dichloro-4-(4-chlorobenzoyl)benzyl)-*1H*-1,2,3-triazole-4-carboxamide (**aa**), to give the compound 5-amino-1-(4-benzoyl-3,5-dichlorobenzyl)-*1H*-1,2,3-triazole-4-carboxamide (**ab**) (83 mg, 25 %) as a yellow solid.**^{1}H-NMR** (300MHz, DMSO-*d$_6$*): δ 7.77-7.73 (m, 3H), 7.62-

7.55 (m, 2H), 7.51 (s, 1H), 7.39 (d, *J*=8.1 Hz, 2H), 6.53 (s, 2H), 5.52 (s, 2H). **^{13}C-NMR** (75MHz, DMSO-*d*$_6$): *δ* 192.24, 164.58, 145.39, 140.89, 136.67, 135.52, 135.01, 131.21, 130.03, 129.67, 127.93, 122.25, 47.51. **HRMS** (APCI): calculated for $C_{17}H_{14}Cl_2N_5O_2$ [M+H]$^+$:390.0519, found: 309.0519.

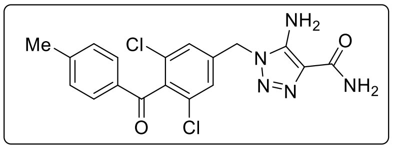

Synthesis of 5-amino-1-(3,5-dichloro-4-(4-methylbenzoyl)benzyl)-*1H*-1,2,3-triazole-4-carboxamide (ac)

According to the method of 5-amino-1-(3,5-dichloro-4-(4-chlorobenzoyl)benzyl)-*1H*-1,2,3-triazole-4-carboxamide (**aa**), to give the compound 5-amino-1-(3,5-dichloro-4-(4-methylbenzoyl)benzyl)-*1H*-1,2,3-triazole-4-carboxamide (ac) (105 mg, 42 %) as a yellow powder. **^{1}H-NMR** (300MHz, DMSO-*d*$_6$): *δ* 7.64 (d, *J*=8.6 Hz, 2H), 7.43 (s, 2H), 7.39 (d, *J*=8.1 Hz, 2H), 6.53 (s, 2H), 5.52 (s, 2H), 2.39 (s, 3H). **^{13}C-NMR** (75MHz, DMSO-*d*$_6$): *δ* 191.65, 164.56, 146.40, 145.36, 140.72, 136.84, 132.64, 131.19, 130.56, 129.80, 127.86, 122.22, 47.50, 21.84. **HRMS** (APCI): calculated for $C_{18}H_{16}Cl_2N_5O_2$ [M+H]$^+$:404.0676, found: 404.0675.

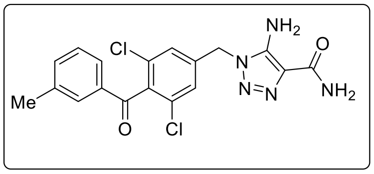

Synthesis of 5-amino-1-(3,5-dichloro-4-(3-methylbenzoyl)benzyl)-*1H*-1,2,3-triazole-4-carboxamide (ad)

According to the method of 5-amino-1-(3,5-dichloro-4-(4-chlorobenzoyl)benzyl)-*1H*-1,2,3-triazole-4-carboxamide (**aa**), to give the compound 5-amino-1-(3,5-dichloro-4-(3-methylbenzoyl)benzyl)-*1H*-1,2,3-triazole-4-carboxamide (**ad**) (94 mg, 27 %) as a yellow solid. **^{1}H-NMR** (300MHz, DMSO-*d*$_6$): *δ* 7.62-7.60 (m, 1H), 7.58-7.54 (m, 1H), 7.52-7.46 (m, 3H), 7.43 (s, 2H), 7.15 (s, 1H), 6.52 (s, 2H), 5.52 (s, 2H), 2.37 (s, 3H).**^{13}C-NMR** (75MHz, DMSO-*d*$_6$): *δ* 192.29, 164.63, 145.42, 140.79, 139.58, 136.82, 136.20, 135.12, 131.23, 129.86, 129.51, 127.89, 127.27, 122.26, 47.55, 21.23. **HRMS** (APCI): calculated for $C_{18}H_{16}Cl_2N_5O_2$ [M+H]$^+$:404.0676, found: 404.0673.

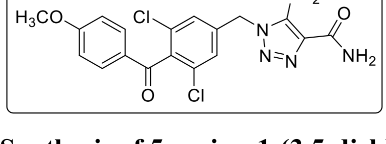

Synthesis of 5-amino-1-(3,5-dichloro-4-(4-methoxybenzoyl)benzyl)-*1H*-1,2,3-triazole-4-carboxamide (ae)

According to the method of 5-amino-1-(3,5-dichloro-4-(4-chlorobenzoyl)benzyl)-*1H*-1,2,3-triazole-4-carboxamide (**aa**), to give the compound 5-amino-1-(3,5-dichloro-4-(4-methoxybenzoyl)benzyl)-*1H*-1,2,3-triazole-4-carboxamide (**ae**) (88 mg, 35 %) as a yellow solid. **^{1}H-NMR** (300MHz, DMSO-*d*$_6$): *δ* 7.70 (d, *J*=9.0

Hz, 2H), 7.43 (s, 2H), 7.10 (d, *J*=9.0 Hz, 2H), 6.77 (s, 2H), 5.52 (s, 2H), 3.85 (s, 3H). **^{13}C-NMR** (75MHz, DMSO-*d$_6$*): *δ* 190.36, 164.98, 162.33, 146.83, 140.33, 137.05, 132.20, 131.25, 128.05, 127.85, 115.31, 56.25, 51.35, 47.60. **HRMS** (APCI): calculated for $C_{18}H_{16}Cl_2N_5O_3$ $[M+H]^+$: 420.0625, found: 420.0629.

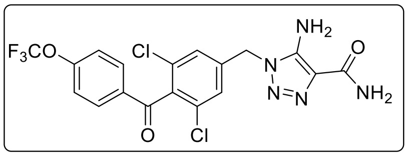

Synthesis of 5-amino-1-(3,5-dichloro-4-(4-(trifluoromethoxy)benzoyl)benzyl)-*1H*-1,2,3-triazole-4-carboxamide (af)

According to the method of 5-amino-1-(3,5-dichloro-4-(4-chlorobenzoyl)benzyl)-*1H*-1,2,3-triazole-4-carboxamide (**aa**), to give the compound 5-amino-1-(3,5-dichloro-4-(4-(trifluoromethoxy)benzoyl)benzyl)- *1H*-1,2,3-triazole-4-carboxamide (**af**) (101 mg, 42 %) as a yellow powder. **^{1}H-NMR** (300MHz, DMSO-*d$_6$*): *δ* 7.91 (dd, *J*=2.0 Hz, 6.8 Hz, 2H), 7.57 (dd, *J*=1.2 Hz, 9.0 Hz, 2H), 7.48 (s, 2H), 7.17 (s, 1H), 6.54 (s, 2H), 5.53 (s, 2H). **^{13}C-NMR** (75MHz, DMSO-*d$_6$*): *δ* 190.53, 164.12, 152.74, 144.92, 140.73, 135.65, 133.19, 131.86, 130.73, 127.60, 121.78, 121.44, z47.05. **HRMS** (APCI): calculated for $C_{18}H_{13}Cl_2F_3N_5O_3$ $[M+H]^+$: 474.0342, found:474.0344.

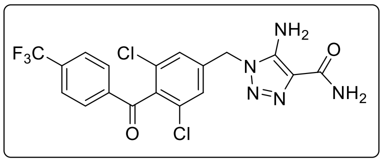

Synthesis of 5-amino-1-(3,5-dichloro-4-(4-(trifluoromethyl)benzoyl)benzyl)-*1H*-1,2,3-triazole-4-carboxamide (ag)

According to the method of 5-amino-1-(3,5-dichloro-4-(4-chlorobenzoyl)benzyl)-*1H*-1,2,3-triazole-4-carboxamide (**aa**), to give the compound 5-amino-1-(3,5-dichloro-4-(4-(trifluoromethyl)benzoyl)benzyl)- *1H*-1,2,3-triazole-4- carboxamide (**ag**) (87 mg, 36 %) as a yellow solid. **^{1}H-NMR** (300MHz, DMSO-*d$_6$*): *δ* 7.98 (s, 4H), 7.49 (s, 2H), 6.53 (s, 2H), 5.54 (s, 2H). **^{13}C-NMR** (75MHz, DMSO-*d$_6$*): *δ* 164.55, 145.37, 137.89, 135.85, 134.69, 131.20, 130.55, 130.49, 128.09, 126.70, 122.22, 121.91, 121.56, 58.06, 47.50. **HRMS** (APCI): calculated for $C_{18}H_{13}Cl_2F_3N_5O_2$ $[M+H]^+$: 458.0393, found: 458.0394.

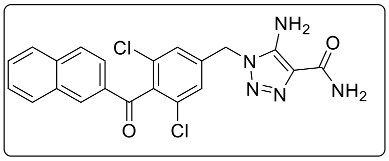

Synthesis of 1-(4-(2-naphthoyl)-3,5-dichlorobenzyl)-5-amino-*1H*-1,2,3-triazole-4-carboxamide (ah)

According to the method of 5-amino-1-(3,5-dichloro-4-(4-chlorobenzoyl)benzyl)-*1H*-1,2,3-triazole-4-

carboxamide (**aa**), to give the compound 1-(4-(2-naphthoyl)-3,5-dichlorobenzyl)-5-amino-*1H*-1,2,3-triazole -4-carboxamide **(ah)** (91 mg, 24 %) as a yellow solid. **^{1}H-NMR** (300MHz, $CDCl_3$): δ 8.31 (d, *J*=3.0 Hz, 1H) 8.16-8.10 (t, *J*= 9.0 Hz, 2H), 8.04 (d, *J*=6.0 Hz, 1H), 7.93 (dd, *J*=3.0, 9.0 Hz, 1H), 7.75-7.70 (m, 1H), 7.65-7.60 (m, 1H), 7.51 (s, 1H), 7.49 (s, 2H), 7.16 (s, 1H), 6.55 (s, 2H), 5.56 (s, 2H). **^{13}C-NMR** (75MHz, $CDCl_3$): δ 192.62, 164.79, 144.88, 137.95, 137.71, 136.35, 132.84, 132.73, 132.50, 132.44, 129.90, 129.43, 129.21, 127.92, 127.10, 126.93, 123.84, 122.74, 48.43. **HRMS** (APCI): calculated for $C_{21}H_{16}Cl_2N_5O_2$ $[M+H]^+$: 440.0676, found:440.0676.

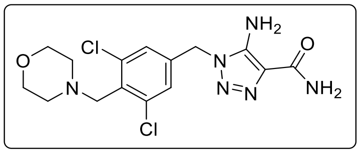

Synthesis of 5-amino-1-(3,5-dichloro-4-(morpholinomethyl)benzyl)-*1H*-1,2,3-triazole-4-carboxamide (ai)

According to the method of 5-amino-1-(3,5-dichloro-4-(4-chlorobenzoyl)benzyl)-*1H*-1,2,3-triazole-4-carboxamide (**aa**), to give the compound 5-amino-1-(3,5-dichloro-4-(morpholinomethyl)benzyl)-*1H*-1,2,3-triazole-4-carboxamide (**ai**) (84 mg, 33 %) as a yellow solid. **^{1}H-NMR** (300MHz, DMSO-d_6): δ 7.28 (s, 2H), 5.42 (s, 2H), 3.63 (s, 2H), 3.49 (t, *J*=4.5 Hz, 4H), 2.43 (t, *J*=4.5 Hz, 4H). **^{13}C-NMR** (75MHz, DMSO-d_6): δ 164.55, 145.25, 138.54, 138.30, 136.60, 133.28, 127.86, 122.15, 66.66, 56.58, 53.58, 47.34. **HRMS** (APCI): calculated for $C_{15}H_{19}Cl_2N_6O_2$ $[M+H]^+$: 385.0941, found: 385.0943.

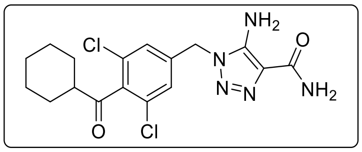

Synthesis of 5-amino-1-(3,5-dichloro-4-(cyclohexanecarbonyl)benzyl)-*1H*-1,2,3-triazole-4-carboxamide (aj)

According to the method of 5-amino-1-(3,5-dichloro-4-(4-chlorobenzoyl)benzyl)-*1H*-1,2,3-triazole-4-carboxamide (**aa**), to give the compound 5-amino-1-(3,5-dichloro-4-(cyclohexanecarbonyl)benzyl)-*1H*-1,2,3-triazole-4-carboxamide (**aj**) (76 mg, 23 %) as a yellow solid. **^{1}H-NMR** (300MHz, DMSO-d_6): δ 7.48 (s, 1H), 7.35 (s, 1H), 7.24 (s, 1H), 7.13 (s, 1H), 6.48 (s, 2H), 5.45 (s, 2H), 1.88-1.57 (m, 5H), 1.41-1.11 (m, 6H). **^{13}C-NMR** (75MHz, DMSO-d_6): δ 204.37, 164.13, 144.88, 144.84, 140.02, 134.26, 129.97, 127.46, 126.27, 121.76, 121.72, 50.52, 47.18, 46.95, 27.59, 25.00. **HRMS** (APCI): calculated for $C_{17}H_{19}Cl_2N_5O_2$ $[M+H]^+$: 396.0989, found: 396.0989.

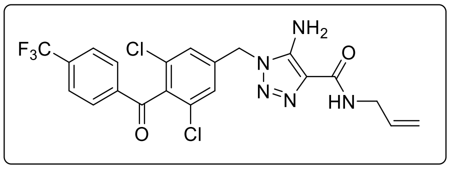

Synthesis of *N*-allyl-5-amino-1-(3,5-dichloro-4-(4-(trifluoromethyl)benzoyl)benzyl)-*1H*-1,2,3-triazole-4-carboxamide (ak)

A solution of *N*-allyl-2-cyanoacetamide (154 mg, 0.80 mmol) in methanol (6.0 mL) at room temperature was treated with sodium ethoxide (56 mg, 0.83 mmol) followed by (4-(azidomethyl)-2,6-dichlorophenyl) (4-(trifluoromethyl)phenyl)methanone (200 mg, 0.53 mmol). The reaction was then stirred for 2h at 50 °C. After cooling to room temperature the reaction was diluted by addition of water and EtOAc, the organic phase separated, the aqueous layer was extracted with EtOAc and the organic phase washed with brine, dried over anhydrous $MgSO_4$, filtered and concentrated. The residue was purified by silica gel column chromatography (CH_2Cl_2/ EtOAc 10/1) to give the compound to give the title compound *N*-allyl-5-amino-1-(3,5-dichloro-4-(4-(trifluoromethyl)benzoyl)benzyl)-1*H*-1,2,3-triazole-4- carboxamide (**ak**) (108 mg, 36 %) as a yellow solid. **^{1}H-NMR** (300MHz, DMSO-*d$_6$*): δ 8.30 (t, *J*=6.0 Hz, 1H), 7.98 (s, 4H), 7.50 (s, 2H), 6.54 (s, 2H), 5.91-5.80 (m, 1H), 5.55 (s, 2H), 5.16-5.02 (m ,2H), 3.85 (t, *J*=6.0 Hz, 2H). **^{13}C-NMR** (75MHz, DMSO-*d$_6$*): δ 191.68, 162.27, 145.18, 141.34, 137.88, 136.21, 135.86, 134.71, 134.29, 131.21, 130.48, 128.10, 127.13, 122.19, 115.36, 47.54, 46.13. **HRMS** (APCI): calculated for $C_{21}H_{17}Cl_2F_3N_5O_2$ $[M+H]^+$:498.0706, found: 498.0705.

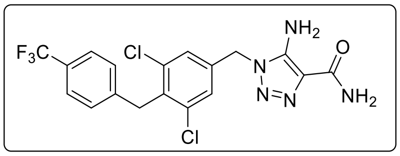

Synthesis of 5-amino-1-(3,5-dichloro-4-(4-(trifluoromethyl)benzyl)benzyl)-*1H*-1,2,3-triazole-4-carboxamide (al)

According to the method of 5-amino-1-(3,5-dichloro-4-(4-chlorobenzoyl)benzyl)-*1H*-1,2,3-triazole-4-carboxamide (**aa**), to give the compound 5-amino-1-(3,5-dichloro-4-(4-(trifluoromethyl)benzyl)benzyl) -*1H*-1,2,3-triazole-4- carboxamide (**al**) (91 mg, 24 %) as a yellow solid. **^{1}H-NMR** (300MHz, DMSO-*d$_6$*): δ 7.65 (d, *J*=8.3 Hz, 2H), 7.50 (s, 1H), 7.38 (s, 2H), 7.31 (d, *J*=8.3 Hz, 2H), 7.15 (s, 2H), 6.48 (s, 2H), 5.43 (2H), 4.23 (s, 2H). **^{13}C-NMR** (75MHz, DMSO-*d$_6$*): δ 164.59, 145.29, 142.86, 138.33, 135.71, 134.96, 134.71, 129.10, 128.09, 126.72, 125.92, 122.19, 47.35, 36.11. **HRMS** (APCI): calculated for $C_{18}H_{15}Cl_2F_3N_5O$ $[M+H]^+$: 444.0601, found:444.0599.

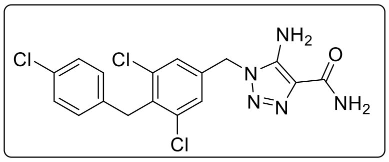

Synthesis of 5-amino-1-(3,5-dichloro-4-(4-chlorobenzyl)benzyl)-*1H*-1,2,3-triazole-4-carboxamide (am)

According to the method of 5-amino-1-(3,5-dichloro-4-(4-chlorobenzoyl)benzyl)-*1H*-1,2,3-triazole-4-carboxamide (**aa**), 5-amino-1-(3,5-dichloro-4-(4-chlorobenzyl)benzyl)-*1H*-1,2,3-triazole-4-carboxamide (**am**) (88 mg, 25 %) as a yellow soild. **^{1}H-NMR** (300MHz, DMSO-d_6): δ 7.50 (s, 1H), 7.36-7.31 (m, 4H), 7.14-7.10 (m, 3H), 6.48 (s, 2H). 5.43 (s, 2H), 4.23 (s, 2H). **^{13}C-NMR** (75MHz, DMSO-d_6): δ 164.59, 145.28, 138.15, 136.95, 135.63, 135.40, 131.47, 130.17, 128.93, 128.05, 122.18, 47.35, 35.60. **HRMS** (APCI): calculated for $C_{17}H_{15}Cl_3N_5O$ $[M+H]^+$: 410.0337, found: 410.0338.

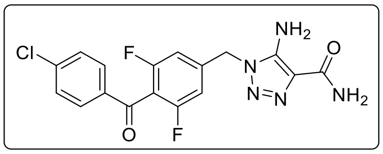

Synthesis of 5-amino-1-(4-(4-chlorobenzoyl)-3,5-difluorobenzyl)-*1H*-1,2,3-triazole-4-carboxamide (an)

According to the method of 5-amino-1-(3,5-dichloro-4-(4-chlorobenzoyl)benzyl)-*1H*-1,2,3-triazole-4-carboxamide (**aa**), to give the compound 5-amino-1-(4-(4-chlorobenzoyl)-3,5-difluorobenzyl)-*1H*-1,2,3-triazole-4-carboxamide (**an**) (76 mg, 23 %) as a yellow solid. **^{1}H-NMR** (300MHz, DMSO-d_6): δ 7.82 (d, J= 8.6 Hz, 2H), 7.65 (d, J= 8.7 Hz, 2H), 7.50 (s,1H), 7.37 (s, 2H), 7.15-7.10 (m ,3H), 6.51 (s, 2H), 5.55 (s, 2H). **^{13}C-NMR** (75MHz, DMSO-d_6): δ 187.53, 164.57, 145.44, 140.43, 135.12, 131.58, 130.00, 129.82, 122.22, 112.02, 111.70, 56.83, 47.87. **HRMS** (APCI): calculated for $C_{17}H_{13}ClF_2N_5O_2$ $[M+H]^+$: 392.0721, found: 392.0714.

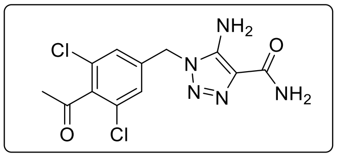

Synthesis of 1-(4-acetyl-3,5-dichlorobenzyl)-5-amino-*1H*-1,2,3-triazole-4-carboxamide (ao)

According to the method of 5-amino-1-(3,5-dichloro-4-(4-chlorobenzoyl)benzyl)-*1H*-1,2,3-triazole-4-carboxamide (**aa**), to give the compound 1-(4-acetyl-3,5-dichlorobenzyl)-5-amino-*1H*-1,2,3-triazole -4-carboxamide (**ao**) (81 mg, 29 %) as a yellow solid. **^{1}H-NMR** (300MHz, DMSO-d_6): δ 7.49 (s, 1H), 7.37 (s, 2H), 7.14 (s, 1H), 6.49 (s, 2H). 5.46 (s, 2H), 2.53 (s, 3H). **^{13}C-NMR** (75MHz, DMSO-d_6): δ 200.03, 164.53, 145.30, 140.40, 139.07, 129.60, 127.85, 122.20, 47.43, 31.45. **HRMS** (APCI): calculated for $C_{12}H_{12}Cl_2N_5O_2$ $[M+H]^+$: 328.0363, found: 328.0362.

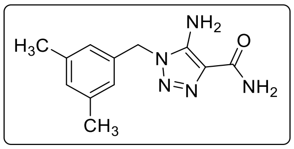

Synthesis of 5-amino-1-(3,5-dimethylbenzyl)-*1H*-1,2,3-triazole-4-carboxamide (ap)

According to the method of 5-amino-1-(3,5-dichloro-4-(4-chlorobenzoyl)benzyl)-*1H*-1,2,3-triazole-4-carboxamide (**aa**), to give the compound **5**-amino-1-(3,5-dimethylbenzyl)-*1H*-1,2,3-triazole-4-carboxamide (**ap**) (57 mg, 27 %) as a yellow solid. **^{1}H-NMR** (300MHz, DMSO-d_6): δ 77.44 (s, 1H), 7.09 (s, 1H), 6.93 (s, 1H), 6.82 (s, 2H), 6.35 (s, 2H), 5.32 (s, 2H). 2.23 (s, 6H). **^{13}C-NMR** (75MHz, DMSO-d_6): ppm δ 164.72, 145.15, 138.08, 136.25, 129.52, 125.53, 124.68, 122.10, 48.67, 21.35. **HRMS** (APCI): calculated for $C_{12}H_{16}N_5O$ $[M+H]^+$: 246.1350, found: 246.1355.

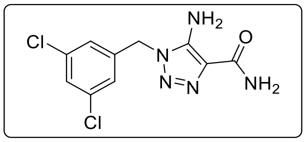

Synthesis of 5-amino-1-(3,5-dichlorobenzyl)-*1H*-1,2,3-triazole-4-carboxamide (aq)

According to the method of 5-amino-1-(3,5-dichloro-4-(4-chlorobenzoyl)benzyl)-*1H*-1,2,3-triazole-4-carboxamide (**aa**), to give the compound 5-amino-1-(3,5-dichlorobenzyl)-*1H*-1,2,3-triazole-4-carboxamide (**aq**) (71 mg, 25 %) as a yellow solid. **^{1}H-NMR** (300MHz, DMSO-d_6): δ 7.59 (t, *J*= 2.4 Hz,1H), 7.48 (s, 1H), 7.25 (d, *J*= 2.4 Hz, 2H), 7.13 (s, 1H). 6.47 (s, 2H), 5.44 (s, 2H). **^{13}C-NMR** (75MHz, DMSO-d_6): ppm δ 164.56, 145.28, 140.47, 134.70, 127.96, 126.71, 122.17, 47.62. **HRMS** (APCI): calculated for $C_{10}H_{10}Cl_2N_5O$ $[M+H]^+$: 286.0257, found: 286.0255.

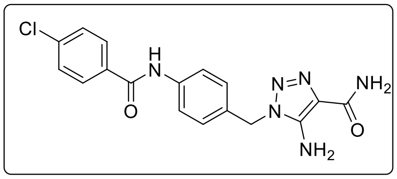

Synthesis of 5-amino-1-(4-(4-chlorobenzamido)benzyl)-*1H*-1,2,3-triazole-4-carboxamide (ba)

According to the method of 5-amino-1-(3,5-dichloro-4-(4-chlorobenzoyl)benzyl)-*1H*-1,2,3-triazole-4-carboxamide (**aa**), to give the compound 5-amino-1-(4-(4-chlorobenzamido)benzyl)-*1H*-1,2,3-triazole-4-carboxamide (**ba**) (76 mg, 27 %) as a yellow solid. **^{1}H-NMR** (300MHz, DMSO-d_6): δ 10.36 (s, 1H), 7.97 (d, *J*=8.4 Hz, 2H), 7.74 (d, *J*=8.4 Hz, 2H), 7.61 (d, *J*=8.6 Hz, 2H), 7.47 (s, 1H), 7.22 (d, *J*=8.6 Hz, 2H), 7.10 (s, 1H), 6.39 (s, 2H), 5.38 (s, 2H). **^{13}C-NMR** (75MHz, DMSO-d_6): ppm δ 164.29, 144.70, 138.50, 136.46, 131.21, 130.01, 129.64, 129.01, 128.47, 127.88, 121.73, 120.53, 48.00. **HRMS** (APCI): calculated for $C_{17}H_{16}ClN_6O_2$ $[M+H]^+$:371.1018, found: 371.1018.

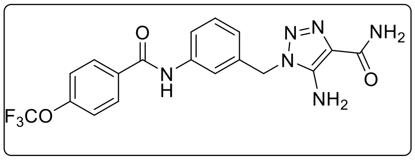

Synthesis of 5-amino-1-(3-(4-(trifluoromethoxy)benzamido)benzyl)-*1H*-1,2,3-triazole-4-carboxamide (bb)

According to the method of 5-amino-1-(3,5-dichloro-4-(4-chlorobenzoyl)benzyl)-*1H*-1,2,3-triazole-4-carboxamide (**aa**), to give the compound 5-amino-1-(3-(4-(trifluoromethoxy)benzamido)benzyl)-*1H*-1,2,3-triazole-4-carboxamide (**bb**) (81 mg, 32 %) as a yellow solid. **^{1}H-NMR** (300MHz, DMSO-d_6): δ 10.43 (s, 1H), 8.11-8.08 (m 2H), 7.54 (s, 1H), 7.52-7.49 (m, 2H), 7.42-7.32 (m, 2H), 7.16 (s, 1H), 6.40 (s, 2H), 5.43 (s, 2H), 4.47 (s, 2H). **^{13}C-NMR** (75MHz, DMSO-d_6): ppm δ 165.01, 151.07, 145.44, 139.94, 137.11, 136.78, 134.57, 130.69, 129.65, 124.39, 123.45, 122.28, 121.27, 120.63, 119.65, 54.27. **HRMS** (APCI): calculated for $C_{18}H_{16}F_3N_6O_3$ $[M+H]^+$:421.1231, found: 421.1231.

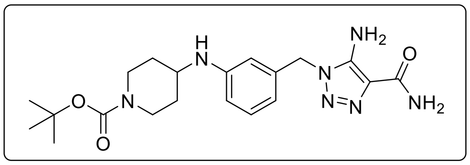

Synthesis of *tert*-butyl 4-((3-((5-amino-4-carbamoyl-*1H*-1,2,3-triazol-1-yl)methyl)phenyl)amino) piperidine-1-carboxylate (bc)

According to the method of 5-amino-1-(3,5-dichloro-4-(4-chlorobenzoyl)benzyl)-*1H*-1,2,3-triazole-4-carboxamide (**aa**), to give the compound *tert*-butyl 4-((3-((5-amino-4-carbamoyl-*1H*-1,2,3-triazol-1-yl)methyl) phenyl)amino) piperidine-1-carboxylate (**bc**) 61mg, 24 %) as a yellow solid. **^{1}H-NMR** (300MHz, DMSO-d_6): δ 7.44 (s, 1H), 7.11-6.97 (m, 2H), 6.51 (d, J= 6.0 Hz, 1H), 6.41-6.30 (m, 4H), 5.60 (d, J= 8.4 Hz, 2H), 5.28 (s, 2H), 4.41 (q, J= 6.8, 14.0 Hz, 1H), 3.86 (d, J= 13.2 Hz, 2H), 3.40-3.29 (m, 2H), 2.95-2.85 (m, 2H), 1.90-1.79 (m, 2H), 1.40 (s, 9H). **^{13}C-NMR** (75MHz, DMSO-d_6): ppm δ 164.33, 153.92, 147.85, 144.79, 136.60, 129.16, 121.64, 114.36, 111.38, 111.29, 54.88, 48.59, 31.42, 28.08. **HRMS** (APCI): calculated for $C_{20}H_{30}N_7O_3$ $[M+H]^+$:416.2405, found:416.2404.

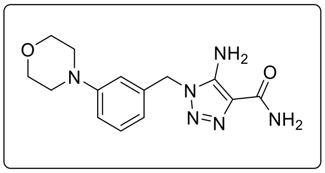

Synthesis of 5-amino-1-(3-morpholinobenzyl)-*1H*-1,2,3-triazole-4-carboxamide (bd)

According to the method of 5-amino-1-(3,5-dichloro-4-(4-chlorobenzoyl)benzyl)-*1H*-1,2,3-triazole-4-carboxamide (**aa**), to give the compound 5-amino-1-(3-morpholinobenzyl)-*1H*-1,2,3-triazole-4-carboxamide (**bd**) (83 mg, 30 %) as a yellow solid. **^{1}H-NMR** (300MHz, DMSO-d_6): δ 7.44 (s, 1H), 7.19 (t, J=7.4 Hz, 1H), 7.08 (s,1H), 6.89 (s, 2H), 6.60 (d, J=8.4 Hz, 1H), 6.36 (s, 2H), 5.33 (s, 2H), 3.73(t, J=5.0 Hz, 4H), 3.07(t, J=5.0 Hz, 4H). **^{13}C-NMR** (75MHz, DMSO-d_6): ppm δ 164.71, 151.64, 145.14, 137.06, 129.70, 122.05, 118.51, 114.84, 114.68, 66.49, 49.08, 48.76. **HRMS** (APCI): calculated for $C_{14}H_{19}N_6O_2$ $[M+H]^+$:303.1564, found: 303.1564.

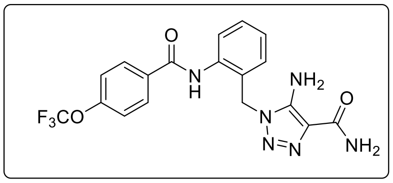

Synthesis of 5-amino-1-(2-(4-(trifluoromethoxy)benzamido)benzyl)-*1H*-1,2,3-triazole-4-carboxamide (be)

According to the method of 5-amino-1-(3,5-dichloro-4-(4-chlorobenzoyl)benzyl)-*1H*-1,2,3-triazole-4-carboxamide (**aa**), to give the compound 5-amino-1-(2-(4-(trifluoromethoxy)benzamido)benzyl)-*1H*-1,2,3 -triazole-4-carboxamide (**be**) (77 mg, 24 %) as a yellow solid. **^{1}H-NMR** (300MHz, DMSO-*d$_6$*): δ 10.30 (s, 1H), 8.16 (dd, *J*=1.9, 6.8 Hz, 2H), 7.56 (d, *J*=8.0 Hz, 2H), 7.50 (d, *J*=6.7 Hz, 2H), 7.37 (t, *J*=7.9 Hz, 1H), 7.24 (t, *J*=7.6 Hz,1H), 7.12 (s, 1H), 6.92 (d, *J*=7.7 Hz, 1H), 6.34(s, 2H), 5.46 (s, 2H). **^{13}C-NMR** (75MHz, DMSO-*d$_6$*): ppm δ 164.93, 164.65, 145.43, 135.85, 133.94, 131.32, 130.65, 128.61, 128.03, 126.96, 126.73, 122.31, 121.24, 45.88. **HRMS** (APCI): calculated for $C_{18}H_{16}F_3N_6O_3$ $[M+H]^+$:421.1231, found:421.1129.

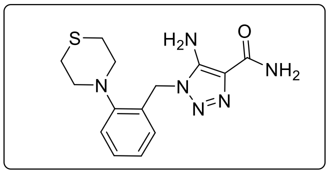

Synthesis of 5-amino-1-(2-thiomorpholinobenzyl)-*1H*-1,2,3-triazole-4-carboxamide (bf)

According to the method of 5-amino-1-(3,5-dichloro-4-(4-chlorobenzoyl)benzyl)-*1H*-1,2,3-triazole-4-carboxamide (**aa**), to give the compound 5-amino-1-(2-thiomorpholinobenzyl)-*1H*-1,2,3-triazole-4- carboxamide (**bf**) (64 mg, 23 %) as a yellow solid. **^{1}H-NMR** (300MHz, DMSO-*d$_6$*): δ 7.47 (s, 1H),7.29-7.19 (m, 2H), 7.12-7.03 (m, 2H), 6.65 (d, *J*=7.4 Hz, 1H), 6.37 (s, 2H), 5.39 (s, 2H), 3.12-3.07 (m, 4H), 2.82-2.77 (m, 4H). **^{13}C-NMR** (75MHz, DMSO-*d$_6$*): ppm δ 164.71, 152.13, 145.70, 131.47, 129.01, 127.86, 124.80, 122.02, 121.66, 55.11, 49.06, 28.05.**HRMS** (APCI): calculated for $C_{14}H_{19}N_6OS$ $[M+H]^+$:319.1336, found: 319.1334.

3.2. Synthesis of CAI derivatives ar-aw

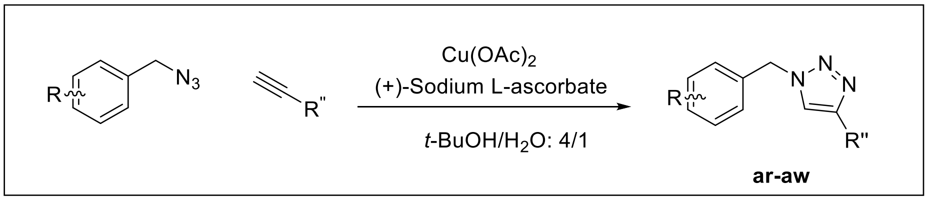

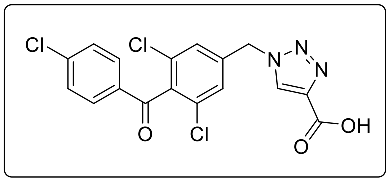

Synthesis of 1-(3,5-dichloro-4-(4-chlorobenzoyl)benzyl)-*1H*-1,2,3-triazole-4-carboxylic acid (ar)

To a solution of propiolic acid (38 μL, 0.62 mmol) and (4-(azidomethyl)-2,6-dichlorophenyl) (4-chlorophenyl) methanone (200 mg, 0.59 mmol) in a *tert*-Butanol/water (5 mL, 4/1) mixture, $Cu(OAc)_2$ (11 mg, 0.06 mmol) and sodium L-ascorbate (23 mg, 0.12 mmol) were added. After stirring for overnight at room temperature, the reaction mixture was diluted with water and extracted with EtOAc, the organic phase separated, the aqueous layer was extracted with EtOAc and the organic phase washed with brine, dried over anhydrous $MgSO_4$, filtered and concentrated in *vacuo*. After the residue was purified by silica gel column chromatography (CH_2Cl_2/ MeOH 50/1, 0.5 % HOAc) to give the compound to give the compound 1-(3,5-dichloro-4-(4-chlorobenzoyl)benzyl)-*1H*-1,2,3-triazole-4-carboxylic acid (**ar**) (173 mg, 72 %) as a light yellow solid. **^{1}H-NMR** (300MHz, DMSO-*d$_6$*): *δ* 13.16 (br s, 1H), 8.88 (s, 1H), 7.78 (dd, *J*= 3.0, 6.0 Hz, 2H), 7.68-7.65 (m, 4H), 5.76 (s, 2H).**^{13}C-NMR** (75MHz, DMSO-*d$_6$*): ppm *δ* 191.23, 162.03, 140.62, 140.39, 133.69, 131.54, 131.30, 130.27, 129.99, 128.93, 51.99. **HRMS** (APCI): calculated for $C_{17}H_{11}Cl_3N_3O_3$ $[M+H]^+$: 409.9861, found: 409.9858.

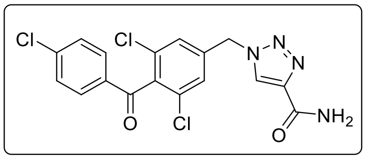

Synthesis of 1-(3,5-dichloro-4-(4-chlorobenzoyl)benzyl)-*1H*-1,2,3-triazole-4-carboxamide (as)

According to the method of 1-(3,5-dichloro-4-(4-chlorobenzoyl)benzyl)-*1H*-1,2,3-triazole-4-carboxylic acid (**ar**), to give the compound 1-(3,5-dichloro-4-(4-chlorobenzoyl)benzyl)-*1H*-1,2,3-triazole-4-carboxamide (**as**) (174 mg, 72 %) as a yellow solid. **^{1}H-NMR** (300MHz, DMSO-*d$_6$*): *δ* 8.73 (s, 1H), 7.89 (s, 1H), 7.79 (dd, *J*= 3.0, 6.0 Hz, 2H), 7.67-7.64 (m, 4H), 7.51 (s, 1H), 5.76 (s, 2H). **^{13}C-NMR** (75MHz, DMSO-*d$_6$*): ppm *δ* 191.22, 161.84, 143.78, 140.62, 140.53, 136.63, 133.69, 131.54, 131.31, 130.27, 128.88, 127.67, 51.99. **HRMS** (APCI): calculated for $C_{17}H_{12}Cl_3N_4O_2$ $[M+H]^+$: 409.0021, found: 409.0018.

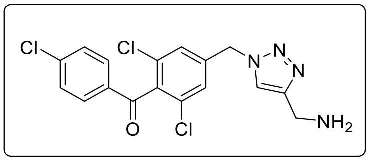

Synthesis of (4-((4-(aminomethyl)-*1H*-1,2,3-triazol-1-yl)methyl)-2,6-dichlorophenyl)(4-chlorophenyl) methanone (at)

According to the method of 1-(3,5-dichloro-4-(4-chlorobenzoyl)benzyl)-*1H*-1,2,3-triazole-4-carboxylic acid (**ar**), to give the compound (4-((4-(aminomethyl)-*1H*-1,2,3-triazol-1-yl)methyl)-2,6-dichlorophenyl) (4-chlorophenyl) methanone (**at**) (165 mg, 71 %) as a yellow solid. **^{1}H-NMR** (300MHz, DMSO-*d$_6$*): *δ* 8.22 (s, 1H), 7.77 (dd, *J*= 3.0, 6.0 Hz, 2H), 7.67 (dd, *J*= 3.0, 6.0 Hz, 2H), 7.61 (s, 2H), 5.74 (s, 2H), 3.97 (s, 2H), 1.01 (t, *J*= 6.8 Hz, 2H). **^{13}C-NMR** (75MHz, DMSO-*d$_6$*): ppm *δ* 191.23, 141.17, 140.64, 136.44, 133.66, 131.50, 131.26, 130.29, 129.43,

128.62, 124.45, 51.63, 35.82. **HRMS** (APCI): calculated for $C_{17}H_{14}Cl_3N_4O$ $[M+H]^+$: 395.0228, found: 395.0228.

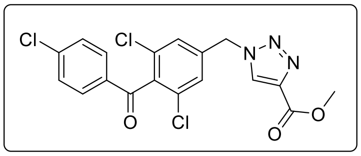

Synthesis of methyl 1-(3,5-dichloro-4-(4-chlorobenzoyl)benzyl)-*1H*-1,2,3-triazole-4-carboxylate (au)

According to the method of 1-(3,5-dichloro-4-(4-chlorobenzoyl)benzyl)-*1H*-1,2,3-triazole-4-carboxylic acid (**ar**), to give the compound methyl 1-(3,5-dichloro-4-(4-chlorobenzoyl)benzyl)-*1H*-1,2,3-triazole-4-carboxylate (**au**) (177 mg, 71 %) as a yellow solid. **^{1}H-NMR** (300MHz, DMSO-d_6): δ 8.99 (s, 1H), 7.78 (dd, *J*= 3.0, 6.0 Hz, 2H), 7.68-7.64 (m, 4H), 5.78 (s, 2H), 3.85 (s, 3H). **^{13}C-NMR** (75MHz, $CDCl_3$): ppm δ 191.22, 161.07, 140.63, 140.25, 139.43, 136.68, 133.68, 131.53, 131.31, 130.26, 128.94, 52.31, 52.08. **HRMS** (APCI): calculated for $C_{18}H_{13}Cl_3N_3O_3$ $[M+H]^+$: 424.0017, found: 424.0014.

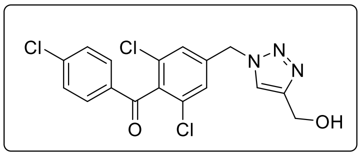

Synthesis of (4-chlorophenyl)(2,6-dichloro-4-((4-(hydroxymethyl)-*1H*-1,2,3-triazol-1-yl)methyl)phenyl) methanone (av)

According to the method of 1-(3,5-dichloro-4-(4-chlorobenzoyl)benzyl)-*1H*-1,2,3-triazole-4-carboxylic acid (**ar**), to give the compound (4-chlorophenyl)(2,6-dichloro-4-((4-(hydroxymethyl)-*1H*-1,2,3-triazol-1-yl) methyl)phenyl) methanone (**av**) (154 mg, 66 %) as a yellow solid. **^{1}H-NMR** (300MHz, DMSO-d_6): δ 8.17 (s, 1H), 7.77 (dd, *J*= 3.0, 6.0 Hz, 2H), 7.67 (dd, *J*= 3.0 6.0 Hz, 2H), 7.60 (s, 2H), 5.70 (s, 2H), 5.20 (t, *J*= 6.0 Hz, 1H), 4.54 (d, *J*= 6.0 Hz, 2H). **^{13}C-NMR** (75MHz, $CDCl_3$): ppm δ 191.23, 149.03, 141.27, 140.61, 136.43, 133.69, 131.51, 131.25, 130.29, 128.59, 123.80, 55.51, 51.60. **HRMS** (APCI): calculated for $C_{17}H_{13}Cl_3N_3O_2$ $[M+H]^+$: 396.0068, found: 396.0065.

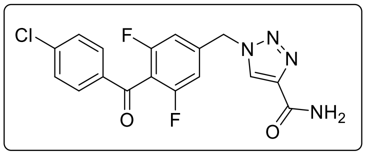

Synthesis of 1-(4-(4-chlorobenzoyl)-3,5-difluorobenzyl)-*1H*-1,2,3-triazole-4-carboxamide (aw)

According to the method of 1-(3,5-dichloro-4-(4-chlorobenzoyl)benzyl)-*1H*-1,2,3-triazole-4-carboxylic acid (**ar**), to give the compound 1-(4-(4-chlorobenzoyl)-3,5-difluorobenzyl)-*1H*-1,2,3-triazole-4-carboxamide (**aw**) (167 mg, 68 %) as a yellow solid. **^{1}H-NMR** (300MHz, DMSO-d_6): δ 8.71 (s, 1H), 7.90 (s, 1H), 7.85 (dd, *J*= 3.0, 6.0 Hz, 2H), 7.67 (dd, *J*= 3.0, 6.0 Hz, 2H). 7.52 (s, 1H), 7.32 (d, *J*= 9.0 Hz, 2H), 5.78 (s, 2H). **^{13}C-NMR** (75MHz,

$CDCl_3$): ppm δ 187.53, 164.62, 161.02, 160.91, 157.70, 145.47, 142.87, 140.44, 135.15, 131.55, 129.97, 122.26, 112.03, 111.70, 47.90.**HRMS** (APCI): calculated for $C_{17}H_{12}ClF_2N_4O_2$ $[M+H]^+$: 377.0612, found: 354.9951.
